# The RNA-binding protein ILF3 binds to transposable element sequences in SINEUP lncRNAs

**DOI:** 10.1096/fj.201901618RR

**Published:** 2019-10-25

**Authors:** Francesca Fasolo, Laura Patrucco, Massimiliano Volpe, Carlotta Bon, Clelia Peano, Flavio Mignone, Piero Carninci, Francesca Persichetti, Claudio Santoro, Silvia Zucchelli, Daniele Sblattero, Remo Sanges, Diego Cotella, Stefano Gustincich

**Affiliations:** * Area of Neuroscience, Scuola Internazionale Superiore di Studi Avanzati (SISSA), Trieste, Italy;; † Department of Health Sciences, Università del Piemonte Orientale, Novara, Italy;; ‡ Central RNA Laboratory, Istituto Italiano di Tecnologia (IIT), Genova, Italy;; § Department of Biology and Evolution of Marine Organisms, Stazione Zoologica Anton Dohrn, Napoli, Italy;; ¶ Institute of Genetic and Biomedical Research (IRGB), National Research Council (CNR), Milan, Italy;; ‖ Humanitas Clinical and Research Center, Rozzano, Italy;; # Department of Sciences and Innovation, Università del Piemonte Orientale, Alessandria, Italy;; ** Division of Genomic Technologies, Riken Center for Life Science Technologies, Yokohama, Japan;; †† Department of Life Sciences, University of Trieste, Trieste, Italy

**Keywords:** RIDome, long noncoding RNA

## Abstract

Transposable elements (TEs) compose about half of the mammalian genome and, as embedded sequences, up to 40% of long noncoding RNA (lncRNA) transcripts. Embedded TEs may represent functional domains within lncRNAs, providing a structured RNA platform for protein interaction. Here we show the interactome profile of the mouse inverted short interspersed nuclear element (SINE) of subfamily B2 (invSINEB2) alone and embedded in antisense (AS) ubiquitin C-terminal hydrolase L1 (Uchl1), an lncRNA that is AS to Uchl1 gene. AS Uchl1 is the representative member of a functional class of AS lncRNAs, named SINEUPs, in which the invSINEB2 acts as effector domain (ED)–enhancing translation of sense protein-coding mRNAs. By using RNA-interacting domainome technology, we identify the IL enhancer-binding factor 3 (ILF3) as a protein partner of AS Uchl1 RNA. We determine that this interaction is mediated by the RNA-binding motif 2 of ILF3 and the invSINEB2. Furthermore, we show that ILF3 is able to bind a free right *Arthrobacter luteus* (Alu) monomer sequence, the embedded TE acting as ED in human SINEUPs. Bioinformatic analysis of Encyclopedia of DNA Elements–enhanced cross-linking immunoprecipitation data reveals that ILF3 binds transcribed human SINE sequences at transcriptome-wide levels. We then demonstrate that the embedded TEs modulate AS Uchl1 RNA nuclear localization to an extent moderately influenced by ILF3. This work unveils the existence of a specific interaction between embedded TEs and an RNA-binding protein, strengthening the model of TEs as functional modules in lncRNAs.—Fasolo, F., Patrucco, L., Volpe, M., Bon, C., Peano, C., Mignone, F., Carninci, P., Persichetti, F., Santoro, C., Zucchelli, S., Sblattero, D., Sanges, R., Cotella, D., Gustincich, S. The RNA-binding protein ILF3 binds to transposable element sequences in SINEUP lncRNAs.

A large portion of the mammalian genome is transcribed, giving rise to a plethora of RNA molecules (1). Among them, long noncoding RNAs (lncRNAs) represent the largest and most heterogeneous class (2–4). lncRNAs are arbitrarily defined as transcripts exceeding 200 nt in length, without evidence of protein-coding capacity. According to the LNCipedia database, the human genome contains more than 118,000 lncRNAs, and this number has increased rapidly (5, 6). Although only a minor portion of lncRNAs have been associated to specific functional roles in cells, it is unanimously accepted that they contribute to gene expression regulation by an array of different mechanisms (7, 8). In eukaryotes, lncRNAs have been found to be prevalent as natural antisense (AS) transcripts (NATs) (9). Specific NATs have been shown to regulate the expression of their sense mRNAs *via* a range of mechanisms that include the inhibition of transcription by steric hindrance of the transcriptional machinery; the repression of expression by competition for transcription factors; the silencing of sense protein expression by RNA interference; or the masking of specific signals on the sense RNA necessary for splicing, stability, or degradation (10, 11).

Regardless of their mode of action, lncRNAs have been proposed to work as modular scaffolds, recruiting and coordinating different effectors through discrete RNA domains with specific secondary structures (12). This model has led to the quest to identify crucial RNA structures within lncRNAs and specific RNA-binding proteins (RBPs) that can mediate their activity.

In this context, transposable elements (TEs) have been proposed as candidate domains that determine the function of lncRNAs (13–16). Previously considered to be junk, TEs are now known to play pivotal roles in shaping genome diversity (17). Interestingly, TEs compose a significant proportion of the lncRNAs, constituting, on average, 40% of the lncRNA nucleotide sequences (18, 19). Recent data demonstrate that embedded TEs are critical modules within lncRNAs that exert their function through protein binding. An embedded *Arthrobacter luteus* (Alu) repeat modulates activity of AS noncoding RNA in the INK4 locus by recruiting protein components of the polycomb repressive complex (20). Binding of Staufen, the double-stranded RBP (dsRBP), and subsequent Staufen-mediated degradation are triggered by the formation of double-stranded RNA (dsRNA) following hybridization between mRNAs and lncRNAs containing complementary Alu fragments (21, 22). Furthermore, heterogeneous ribonucleoprotein particle (hnRNP) C and TAR DNA-binding protein 43 (TDP-43) were shown to bind embedded Alu sequences preferentially in the inverted orientation (23, 24). By using cross-linking immunoprecipitation (CLIP) sequencing, human antigen R and ATP-dependent RNA helicase UPF1 were identified as additional RBPs for inverted Alu sequences that regulate lncRNAs abundance and splicing (25).

One of the key features of genomes’ organization is that most genes share their genomic region with another gene on the opposite filament, forming sense-AS (S/AS) pairs (2, 26). Almost 70% of protein-encoding genes present an AS lncRNA on the opposite strand (26). In a growing number of cases, AS lncRNAs have been shown to be required for proper regulation of coding genes, carrying genetic information that acts at distinct regulatory levels (16, 27, 28).

We previously showed that the mouse lncRNA AS ubiquitin C-terminal hydrolase L1 (Uchl1) can enhance translation of sense protein-coding Uchl1 mRNA through the activity of an embedded TE of the short interspersed nuclear element (SINE) B2 type (13). AS Uchl1 function depends on 2 RNA domains: a 5′ overlapping sequence to the sense transcript that drives the specificity of action and is thus referred to as the binding domain (BD) and an embedded inverted SINE of subfamily B2 (invSINEB2) in the nonoverlapping region, which represents the effector domain (ED) and confers translation-enhancing activity (**Fig. 1*A***). In the nonoverlapping sequence, AS Uchl1 also contains a partial Alu element that is not required for translation up-regulation activity and whose exact function is presently unknown. In physiologic conditions, AS Uchl1 RNA accumulates in the nucleus of neurons, whereas, upon stress, it shuttles into the cytoplasm (13). AS Uchl1 is the representative member of a new functional class of lncRNAs, named SINEUPs, because they rely on a SINEB2 to up-regulate translation and share the combination of BD and ED (16, 29). Several natural SINEUPs have been identified in mouse (13, 16). Although SINEB2 sequences are not present in the human transcriptome, we recently showed that human SINEUPs take advantage of the embedded free right Alu monomer (FRAM) repeat element, which functions as an ED in AS lncRNAs transcripts (30). It is noteworthy that, by manipulating AS Uchl1 BD, synthetic SINEUPs with invSINEB2 or FRAM EDs can be generated to act as translation enhancers of targets of choice (29, 31–33). Although the molecular mechanisms underlying SINEUP subcellular localization and activity remain unclear, SINEUPs are an ideal model to study the relative contribution of pairing and secondary structures in lncRNAs function. In this context, we have recently showed that the invSINEB2 structure exhibits several internal loops and hairpins that may serve as structural motifs for specific recognition by unknown partner molecules (34, 35). Furthermore, given the functional conservation between 2 apparently unrelated embedded TEs, the mouse invSINEB2 and the human FRAM, any common protein partners may strengthen the hypothesis they are acting as convergent functional domains.

**Figure 1:**
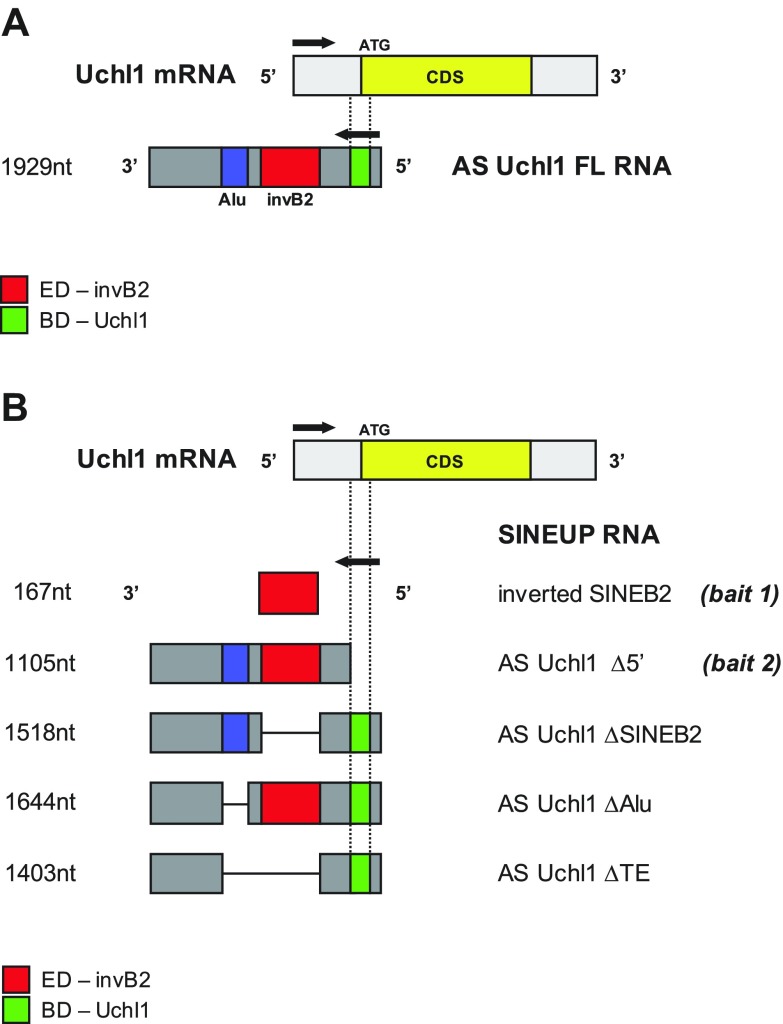
Scheme of SINEUP AS Uchl1 constructs. *A*) The FL clone for AS Uchl1 is shown. The overlapping region with sense Uchl1 mRNA, representing the BD (green), spans 40 nt of Uchl1 5′UTR (gray) and 33 nt of the CDS (yellow). The invSINEB2 is the ED (red) of SINEUP AS Uchl1. *B*) AS Uchl1 mutants are schematically depicted. The invSINEB2 contained in AS Uchl1 and the mutant lacking the BD (AS Uchl1 Δ5′) have been employed as baits in phage display selection. Deletion mutants of TEs have been employed for functional studies. AS Uchl1 ΔSINEB2 and ΔAlu lack the embedded invSINEB2 or Alu, respectively. AS Uchl1 ΔTE is deprived of both repeats. CDS, coding sequence.

Here, we identify proteins that interact with the invSINEB2 of AS Uchl1. To this end, we employ RNA-interacting domainome (RIDome), a high-throughput interaction discovery platform that combines the selection of a phage cDNA library displaying filtered open reading frames (ORFs) with next-generation sequencing (NGS) (36) (outlined in Supplemental Fig. S1). In brief, a phage library of human ORFs is challenged with a biotinylated RNA bait through multiple cycles of selection and amplification. ORF inserts are collected from the selected phages and sequenced by NGS, and the corresponding genes are ranked according to read frequency. High-scoring ORFs indicate the effective interaction with the RNA bait and can be easily rescued from the phage library by inverse PCR. The interaction with the target RNA can be then validated *in vitro* [*e.g.*, by ELISA- and surface plasmon resonance (SPR)–based assays] and with functional assays in cell culture.

We find that the dsRBP IL enhancer-binding factor 3 (ILF3) is an interacting partner of AS Uchl1. This interaction specifically requires one of the 2 dsRNA-binding motifs (dsRBMs) of ILF3 and the invSINEB2 sequence in AS Uchl1. ILF3 also binds FRAM sequences, the embedded TEs in human SINEUPs. By bioinformatics analysis of enhanced CLIP (eCLIP) data for ILF3 from the Encyclopedia of DNA Elements (ENCODE), we confirm that this RBP is a major interacting protein of Alu sequences in human. In addition, we also demonstrate that the embedded TEs modulate AS Uchl1 RNA nuclear localization to an extent moderately influenced by ILF3.

## MATERIALS AND METHODS

### Constructs

Plasmids expressing AS Uchl1 full-length (FL), AS Uchl1 ΔB2, AS Uchl1 ΔAlu, and AS Uchl1 ΔTE (previously referred to as ΔTOT) were prepared as previously described in Carrieri *et al*. (13).

### *In vitro* RNA synthesis and biotinylation

RNA baits used for biopanning experiments and successive ELISA-based assays were synthesized by *in vitro* transcription (MegaScript T7 Transcription Kit; Thermo Fisher Scientific, Waltham, MA, USA). Template DNAs were prepared by PCR using specific primer pairs in which the forward oligonucleotide was tailed with a T7 RNA polymerase minimal promoter. Synthesized RNAs were analyzed by electrophoresis, purified (MegaClear Kit; Thermo Fisher Scientific), quantified by spectrophotometry, and biotinylated at the 3′ end (Pierce RNA 3′ End Biotinylation Kit; Thermo Fisher Scientific). RNA samples were stored at −80°C until use.

### Biopanning procedures

The ORF phage library used in this study, as well the entire procedure to produce and rescue phagemids, has been previously described (36–38). For biopanning experiments, phage particles were suspended in PBS buffer at a concentration of 10^11^ colony-forming units per microliter, and for each selection we used 10^12^ phages. Selections were done using 2 SINEUP-related RNA baits (shown in Fig. 1): AS Uchl1 Δ5′ (the lncRNA AS Uchl1 sequence, depleted of the 73 bp of overlap; ∼1100 nt) and invSINEB2 (the sequence corresponding to the invSINEB2, embedded in AS Uchl1; ∼170 nt).

Each selection experiment was preceded by a preclearing step of subtracting from the library those phages that would unspecifically bind either the magnetic particles or the plastic tube. It was conducted as follows: 20 μl of streptavidin-coated magnetic beads (New England Biolabs, Ipswich, MA, USA) were washed in 10 mM Tris HCl pH 8.0, 1 mM EDTA, 250 mM NaCl, 0.5% Triton X-100 (TENT buffer) and then incubated with 10^12^ phages in 100 μl of TENT buffer for 30 min at room temperature. Beads were then removed with a magnet, and unbound phages were recovered and used for selection.

For biopanning experiments, the RNA baits were diluted to 30 nM in TENT buffer containing 100 U/μl of the RNAse inhibitor Superase-In (Thermo Fisher Scientific); then 100 μl (3 pmol) were added to 20 μl of streptavidin magnetic beads and incubated for 20 min at room temperature. Selections were performed using 2 protocols that differ in the competitor used: single-stranded DNA (ssDNA) from herring sperm or tRNA from *Escherichia coli*. The beads were washed 3 times in TENT buffer; then phages from the precleared libraries were added to the RNA-conjugated beads and incubated for 45 min at room temperature in the presence of 1 μg/μl of ssDNA or tRNA. Beads were then washed extensively in TENT buffer. Bound phages were eluted by a treatment with RNAse A (10 μg/ml in 10 mM Tris pH 8.0, 1 mM EDTA, 15 mM NaCl) for 2 min at room temperature; then the supernatant containing the phages released from beads was used to infect 2 ml of *E. coli* DH5α for 45 min at 37°C. The eluted phage pool was amplified in DH5α cells, and the procedure was repeated for a second round of selection; the stringency of selection was enhanced by increasing the number of washing steps. To avoid an excessive restriction in the output diversity, only 2 cycles of selection were performed for all protocols. After the second round of selection, colonies growing on agar plates were harvested, and plasmid DNA was isolated by standard miniprep procedure. cDNA inserts were PCR-amplified with barcoded molecular identifier–tagged primers and sequenced with an Illumina SmartSeq platform (Illumina, San Diego, CA, USA).

### Bioinformatics analysis of RIDome

Sequences were processed with the NGS Transcriptome Profile Explorer (NGS-Trex) system (*https://www.ngs-trex.disit.unipmn.it/Trex/cms/*) as previously described (36, 39). Briefly, sequences were mapped onto the human genome (U.S. National Center for Biotechnology Information Build 36) using genomic mapping (GMAP) software, and matching sequences were compared with annotated genes. Each gene was then ranked according to the number of supporting sequences (defined as coverage). For genes present in both the selected libraries and in a reference [nonselected (NS)] library, the fold enrichment was also calculated. By using the “differentially expressed genes” tool, it is indeed possible to query results for differentially represented genes between 2 or more data sets. This tool provides a list of differentially expressed genes within the selected libraries compared with the reference. For each differentially expressed gene, the tool provides the number of reads supporting the gene in the reference (ref count), the number of reads supporting the gene in the other samples (other count), the *P* value evaluating the statistical significance of the differential expression, and the fold change (enrichment).

### Bioinformatics analysis of eCLIP data

Human eCLIP data for ILF3 were downloaded from the ENCODE project (40–42) for HepG2 (*https://www.encodeproject.org/experiments/ENCSR786TSC/*) and K562 cell lines (*https://www.encodeproject.org/experiments/ENCSR438KWZ/*). For each cell line 2 replicate experiments were performed. We downloaded the following inputs (normalized bed narrowPeak files): ENCFF340GPD (*https://www.encodeproject.org/files/ENCFF340GPD/*), ENCFF841BJF (*https://www.encodeproject.org/files/ENCFF841BJF/*), ENCFF353RQP (*https://www.encodeproject.org/files/ENCFF353RQP/*), and ENCFF623LPT (*https://www.encodeproject.org/files/ENCFF623LPT/*).

The files contain locations of peaks associated to ILF3 bindings mapped on the human genome (assembly GRCh38) and their enrichment with respect to the input. Peaks were annotated, also keeping in account the strand. Information on the protocols and methods used to produce these data is openly available on the ENCODE project website. Human gene annotations (assembly GRCh38) in GFF3 format were downloaded from Ensembl (43) (*https://useast.ensembl.org/index.html*) and were relative to the Ensembl v.83. Repetitive element annotations relative to the GRCh38 assembly were obtained from the RepeatMasker (44) file transfer protocol site (*http://www.repeatmasker.org/*).

We selected only peaks showing an enrichment value of *P* < 0.05 in both replicates of a given cell line using R and bedtools (45) (v2.26.0, parameters: -u). A custom-made script was written in R (46) (v.3.3.2) making use of bedtools with the aim to uniquely classify each ILF3 peak to overlap with specific genomic features (genes and repeats). Each peak has been classified as belonging to a single class with respect to the closest overlapping or flanking gene. In cases in which peaks could be assigned to more than 1 class, we have used the following priority: coding exon concordant > noncoding exon concordant > coding intron concordant > noncoding intron concordant > coding discordant > noncoding discordant > intergenic. The terms “concordant” and “discordant” indicate whether the annotated strand of the peak is in the same orientation of the overlapping transcript. Plots were produced using the R libraries ggplot2 (47) (v.2.2.1) and cowplot (48) (v.0.7.0). The overlaps found between ILF3 peaks and the genomic features analyzed were visualized and inspected on the Integrative Genomics Viewer (49) (v.2.3.92). Randomization analyses were performed after obtaining the replicate common peaks data set for HepG2 and K562. Each ILF3 peak from the 2 cell lines was randomized 100 times using bedtools (-noOverlapping; -excl). Comparisons between proportions of real and randomized peaks were performed in R using Fisher’s exact test, and the *P* value was corrected using the false discovery rate method.

### Rescue of phagemid clones and subcloning into a pGEX vector

Phagemids clones were rescued from the selected libraries by inverse PCR as previously described (36, 37). Briefly, a pair of specific back-to-back outward primers was designed for each of the tested genes, centering on the nucleotide region identified by the overlapping reads. For each sample, 50 ng of the phagemid DNA minipreps were used as template, and inverse PCR reactions were performed with a Phusion High-Fidelity DNA Polymerase (Thermo Fisher Scientific). PCR products were purified from agarose gel, phosphorylated with T4 polynucleotide kinase, ligated by T4 DNA ligase, and transformed into *E. coli* DH5αF′. Transformants were screened by colony PCR and verified by Sanger sequencing.

For the bacterial expression of glutathione *S*-transferase (GST) fusion products, ORF fragments were excised from the phagemid DNA with the restriction endonucleases *Pte*I and *Nhe*I (Thermo Fischer Scientific), subcloned into a custom-designed pGEX-Flag expression vector (36), and grown in a minifermenter as previously described in Deantonio *et al*. (50). The vector harbors a Flag epitope tag (DYKDDDDK) for the C-terminal tagging of expressed proteins.

### GST fusion protein expression and purification

ORF fragments subcloned in pGEX-Flag were transformed into *E. coli* BL21(DE3) cells. Bacterial cultures (100 ml) were grown at 28°C until optical density at 600 nm reached 0.5 and then induced with 1 mM isopropyl-β-D-thiogalactoside (IPTG)for 3 h. Bacteria were collected by centrifugation, and pellets were suspended in lysis buffer (PBS containing 1% Triton X-100, 200 µg/ml lysozyme, 20 µg/ml DNAse, protease inhibitors), incubated at 4°C for 30 min, and sonicated for 2–3 min. Cell debris was removed by centrifugation and supernatants combined with glutathione-agarose beads (MilliporeSigma, Burlington, MA, USA) at 4°C for 60 min under gentle rotation. After 3 washes in PBS–Tween 0.1% followed by 3 more washes in PBS, GST fusion proteins were eluted in elution buffer (50 mM reduced glutathione, 100 mM NaCl, pH 8.0). Proteins were dialyzed against PBS and checked for purity and concentration by SDS-PAGE. Quantitative densitometry of Coomassie Blue–stained proteins was calculated with ImageJ software (National Institutes of Health, Bethesda, MD, USA) (51) using bovine serum albumin (BSA) as a reference for protein quantification. GST fusion protein integrity was determined by Western blotting using 2 different monoclonal antibodies, targeting GST (clone GST-2; MilliporeSigma) and Flag (clone M2; MilliporeSigma), respectively.

### ELISA

Screening of selected clones in ELISA-based assays, either in the phage format or as soluble GST fusion polypeptides, was performed according to protocols previously described in Patrucco *et al*. (36) with some modifications. Briefly, phage ELISA was performed with Microlon plates (Greiner Bio-One, Kremsmünster, Austria) coated overnight at 4°C with 10 μg/ml streptavidin. After blocking and rinsing wells in TENT buffer, biotinylated RNA transcripts (5 pmol/well, diluted in 100 µl TENT buffer implemented with RNAse inhibitors) were captured on the plates. Phage-containing supernatants of individual clones, diluted 1:1 in TENT buffer with RNAse inhibitors, were added to the wells and incubated for 45 min. Following 3 washing steps, incubation with horseradish peroxidase (HRP)–conjugated anti-M13 monoclonal antibody (GE Healthcare, Waukesha, WI, USA) for 60 min at room temperature was carried out. Signal was revealed with 3,3′,5,5′-tetramethylbenzidine and read at 450 nm using a Victor X4 Multilabel Plate Reader (PerkinElmer, Waltham, MA, USA). ELISA on soluble GST fusion polypeptides was performed similarly as above. After coating and capturing the RNA transcripts, wells were subsequently incubated 60 min at room temperature with the purified proteins, extensively washed in TENT buffer, and again incubated 60 min with a mouse monoclonal anti-GST antibody (clone GST-2; MilliporeSigma) 1:5000 in TENT buffer. Following 1-h incubation with an HRP-conjugated secondary antibody (MilliporeSigma), the signal generated by RNA-protein binding was detected as described above.

### Affinity measurements

The dynamics of SINEUP-ILF3 interactions were characterized by SPR using a Biacore T100 instrument (GE Healthcare) as previously described in Patrucco *et al*. (36). The biotinylated invSINEB2 RNA was immobilized on streptavidin-coated sensor chips (Series S Sensor Chip SA; GE Healthcare). RNA was diluted to a final concentration of 1 μM in 10 mM HEPES and150 mM NaCl, pH 7.4 (HBS-N buffer, GE Healthcare), followed by heating at 80°C for 10 min and cooling to room temperature. The sample was then diluted 500-fold in running buffer (10 mM HEPES, pH 7.4, 150 mM NaCl, 1 mM DTT, 0.025% surfactant P20; GE Healthcare) and injected over the sensor chip surface at 5 µl/min at 25°C to generate an ∼150 response unit.

GST-dsRBM2 was serially diluted in running buffer to the concentrations 300–3.7 nM and injected at 25°C at a flow rate of 30 µl/min for 2 min. Analysis were performed in duplicate, and any background signal from a streptavidin-only reference flow cell was subtracted from every data set.

### Cell culture and transfections

Human embryonic kidney (HEK) 293T/17 cells were obtained from American Type Culture Collection (ATCC-CRL-11268) and cultured in DMEM (Thermo Fisher Scientific) supplemented with 10% fetal bovine serum (FBS; MilliporeSigma), penicillin, and streptomycin.

For RNA immunorecipitation (RNA-IP) experiments, 2.5 × 10^6^ HEK 293T/17 cells were plated in 10-cm dishes and transfected with AS Uchl1 FL plasmid using FuGene HD Transfection Reagent (Promega, Madison, WI, USA), following the manufacturer’s instructions. RNA and proteins were extracted from the same transfection in each replica.

For nucleocytoplasmic fractionation experiments, 4 × 10^5^ cells were plated in 6-well plates and transfected with AS Uchl1 FL, AS Uchl1 ΔB2, AS Uchl1 ΔAlu, or AS Uchl1 ΔTE.

### RNA-IP

Stock solutions were prepared with RNase-free water (treated with diethylpyrocarbonate). Lysis and wash buffers were prepared fresh and kept on ice; all steps, including centrifugation, were performed at 4°C. Forty-eight hours following transfection, cells were washed with PBS, collected by gentle scraping, and centrifuged. Pellets were washed twice with PBS, and cells were fixed in 1% formaldehyde (Mallinckrodt Pharmaceuticals, Dublin, Ireland) in PBS for 10 min at room temperature with slow mixing and then quenched in 0.25 M glycine (pH 7) at room temperature for 5 min. Cells were subsequently harvested by centrifugation at 3000 rpm for 4 min and washed twice with ice-cold PBS. One hundred microliters of sheep anti-mouse magnetic beads (Dynabeads M-280; Thermo Fisher Scientific) were washed 3 times in washing buffer (PBS, 0.1% BSA), blocked with 3 washes in 0.5% BSA, and finally washed twice in RIP lysis buffer (25 mM Tris HCl pH 7.4, 150 mM KCl, 0.5% Igepal CA-630, 5 mM MgCl_2_, 0.5 mM DTT, protease inhibitors, and 20 U/ml Superase RNA inhibitors). Coating with antibody or control IgG was carried out by overnight incubation of blocked beads with 20 μg of anti-ILF3 antibody (612154; BD Biosciences, San Jose, CA, USA) or 20 μg of mouse IgG (as a control) in a final volume of 180 μl. Lysis was performed using 1 ml RIP lysis buffer. Lysates were solubilized by sonication with 2 short pulses (15 s). Between the 2 cycles, samples were kept on ice for at least 2 min. Insoluble material was removed by centrifugation at 13,000 rpm for 10 min. Total lysate was precleared *via* incubation with 100 μl of uncoated blocked beads for 30 min at 4°C with gentle rotation. After recovery from beads, total lysate was split and incubated with specific antibody or control IgG-coated beads overnight on a rotary platform at 4°C. One-twentieth of total precleared lysate was kept before splitting as immunoprecipitation input. Bead-antibody-lysate complexes were washed 6 times (5 min the first and last wash, 1 min the remaining washes) in a cold room. For reversal of cross-linking and elution, beads containing the immunoprecipitation samples were resuspended in 100 μl of elution buffer (50 mM Tris-Cl pH 7.0, 5 mM EDTA, 10 mM DTT, and 1% SDS) and incubated at 70°C for 45 min. Supernatants were recovered and resuspended in 1 ml of Trizol (Thermo Fisher Scientific), and both RNA and proteins were extracted according to the manufacturer’s instructions.

### RNA isolation, reverse transcription, and real-time quantitative PCR

RNA was extracted using Trizol reagent (Thermo Fisher Scientific) according to the manufacturer’s instructions. RNA was eluted and treated with Turbo DNA-Free Kit (Thermo Fisher Scientific) for 15 min at 37°C to avoid plasmid DNA contamination. RNA quality was finally checked on a formaldehyde agarose gel.

cDNA was prepared from 250 ng of purified RNA using iScript cDNA Synthesis Kit (Bio-Rad, Hercules, CA, USA) according to the manufacturer’s instructions. For RNA-IP experiments, equal volumes of DNAse-treated RNA samples were used for reverse transcription. To monitor the efficiency of DNAse treatment, an equal amount of each RNA sample was retrotranscribed in the absence of reverse transcriptase.

Real-time quantitative PCR reaction was performed on diluted cDNA (1:2.5) using Sybr-Green PCR Master Mix (Bio-Rad) and an iCycler IQ Real-Time PCR System (Bio-Rad). In RNA-IP experiments, undiluted cDNA was used as real-time quantitative PCR input.

Oligonuclotide sequences of primers for detection of glyceraldehyde 3-phosphate dehydrogenase (GAPDH) and AS Uchl1, ubiquitin C (UBC), and precursor rRNA (pre-rRNA) were as previously described (13, 52 and 53). The cytochrome B (*CytB*) gene was amplified using the forward primer, 5′-CAATGGCGCCTCAATATTCT-3′, and the reverse primer, 5′-AATGTATGGGTGGCGGATA-3′. Amplified transcripts were quantified using the comparative *C_t_* method, and relative gene expression was calculated with the ΔΔ*C_t_* method (54).

### Western blot

For Western blot analysis, cell pellets were directly dissolved in Laemmli sample buffer. For RNA-IP experiments, ILF3 immunoprecipitation efficiency was monitored by loading the whole fraction of proteins recovered from the organic phase after Trizol extraction, following resuspension in Laemmli sample buffer. All lysates were briefly sonicated, boiled, and loaded on 10% polyacrylamide gels. Immunoblotting was performed with the following primary antibodies: anti-ILF3 (612154; BD Biosciences), 1:500 overnight, and anti–β-actin (A5441; MilliporeSigma), 1:2000. Signals were revealed after incubation with HRP secondary antibodies (Agilent Technologies, Santa Clara, CA, USA) 1:1000 for 1 h at room temperature, in combination with ECL (GE Healthcare). Image detection was performed with Alliance LD2-77WL system (Uvitec, Cambridge, United Kingdom). Image quantification was done using ImageJ software.

### Cell fractionation

Nucleocytoplasmic fractionation was performed as previously described in ref. 55. Fractions were extracted at 48 h post-transfection, and RNA was isolated using Trizol reagent (Thermo Fisher Scientific) following the manufacturer’s instructions. RNA was eluted and treated with Turbo DNAse (Thermo Fisher Scientific). The purity of the nuclear and cytoplasmic fractions was confirmed by real-time quantitative PCR on GAPDH or CytB and pre-rRNA, respectively.

### ILF3 knockdown

HEK 293T/17 cells (4 × 10^5^) were harvested on a 6-well plate and cotransfected with 4 μg of AS Uchl1 plasmid and 4 μg of ILF3 small interfering RNA (siRNA) (Mission esiRNA, mouse ILF3; MilliporeSigma) or control siRNA (All Stars Negative Control siRNA; Qiagen, Germantown, MD, USA) with 10 μl of Lipofectamine 2000 (Thermo Fisher Scientific) in serum-free DMEM with no antibiotics. After 24 h, a second round of transfection was performed, using 2 μg of both plasmid and siRNA. On the following day, medium was changed with 10% FBS-DMEM. At 48 h from the second transfection, cells were collected for fractionation. One-twentieth of the total cells was suspended in Laemmli sample buffer for Western blot analysis of ILF3 protein levels in silenced and control cells. Nucleocytoplasmic fractionation was performed as previously described, and cell fractions were suspended in 1 ml of Trizol.

### Immunofluorescence microscopy

Cells were fixed in 4% paraformaldehyde in PBS for 10 min at room temperature, washed twice in PBS, and treated with glycine 0.1 M in PBS for 5 min. Following 2 more washes in PBS, fixed cells were permeabilized with 0.1% Triton X-100 for 4 min at room temperature and blocked with 0.2% BSA, 1% FBS, and 0.1% Triton in PBS for 5 min. Cells were subsequently incubated 90 min with anti-ILF3 (BD Bioscience) 1:50 in blocking solution, washed in PBS 3 times, and finally stained with AlexaFluor 488– or AlexaFluor 594–labeled anti-mouse or anti-rabbit secondary antibodies (Thermo Fisher Scientific), 1:250 in blocking buffer. Nuclei were visualized with DAPI (1 μg/ml). Anti–DJ-1 1:250 (56) was used to counterstain cell cytoplasm. Images were captured with a confocal microscope (Leica TCS SP2; Leica Microsystems, Buffalo Grove, IL, USA).

### Statistical analysis

All data are expressed as means ± sd for *n* ≥ 3 replicas. Statistical analysis was performed using Excel software. Statistically significant differences were assessed by a Student’s *t* test. Values of *P* < 0.05 were considered significant.

## RESULTS

### Identification of ILF3 as a SINEUP-interacting protein

To identify proteins that interact with natural SINEUP lncRNAs, we employed RIDome (36). The typical outcome of this approach is a list of genes representing putative interacting proteins, ranked based on their enrichment following selection, that will direct subsequent analyses and validation of the best candidates.

The library used in this study has been already described in our previous work (37). In brief, it was constructed with cDNAs from different human cell types (mainly from colon, lung, and pancreas). In the filtering step, cDNA was fragmented into a calibrated size of 100–600 bases and cloned into a vector that allows selection of ORFs that are in the correct frame and fold efficiently in *E. coli*. With respect to canonical FL cDNA phage libraries, this approach has the advantage of generating a normalized library of protein domains (the Domainome) that are homogeneous in terms of peptide length and sequence coverage. It is noteworthy that despite the fact the library derives from only 3 human organs, almost all annotated RBPs and transcription factors are represented by at least 1 read (36). Therefore, the library can be considered universal and, as such, can be used as a tool for the initial identification of proteins interacting with any biomacromolecule of interest (protein, RNA, DNA, *etc.*), regardless of its tissue or organism of origin. Selections were performed using two 3′-biotinylated, *in vitro*–transcribed RNA baits (Fig. 1*B*): AS Uchl1 Δ5′ (corresponding to the mouse AS Uchl1 lncRNA originally discovered by us, in which the 73 nt–long BD was deleted) and the invSINEB2 of AS Uchl1 (the sequence corresponding to the ED alone, ∼170 nt). We avoided the use of FL AS Uchl1 because its function requires the formation of a dsRNA sequence. The reproduction of paired S/AS transcripts as baits in an *in vitro* assay would be challenging. Selections were performed in the presence of tRNA or ssDNA, added as competitors in biopanning solutions to prevent nonspecific binding of the bait. After 2 cycles of selections, phagemid DNA was extracted from the eluted phage pool, and ORF inserts were sequenced on an Illumina platform. We analyzed ∼100,000 reads from each selected library with the NGS-Trex system (37, 39). **Table 1** shows a summary of the sequencing analysis. Sequences matching annotated genes were first ranked according to the number of supporting reads, and genes represented by <20 reads in the selected libraries and by <4 reads in the NS library were considered background noise of the phage selection and thus discarded.

**TABLE 1:** Summary of NGS results

Bait	Competitor	Total reads (*n*)	Mapping reads (*n*)	Mean length (nt)	Genes (*n*)	Genes that met threshold (*n*)
invSINEB2	tRNA	115,501	96,403	201	5255	218
invSINEB2	ssDNA	89,017	71,329	235	5129	198
AS Uchl1 Δ5′	ssDNA	94,695	74,686	258	3938	95
AS Uchl1 Δ5′	tRNA	137,116	110,929	241	5655	295
NS	N/A	155,880	85,448	113	8128	3803

For each selection, the total number of reads, mapped reads, and their mean length are reported. The number of selected genes is shown as well. Arbitrary parameters were applied to narrow the number of selected genes. Threshold was fixed to >4 reads and >20 reads for nonsignificant and selected libraries, respectively. N/A, not applicable.

**Figure 2:**
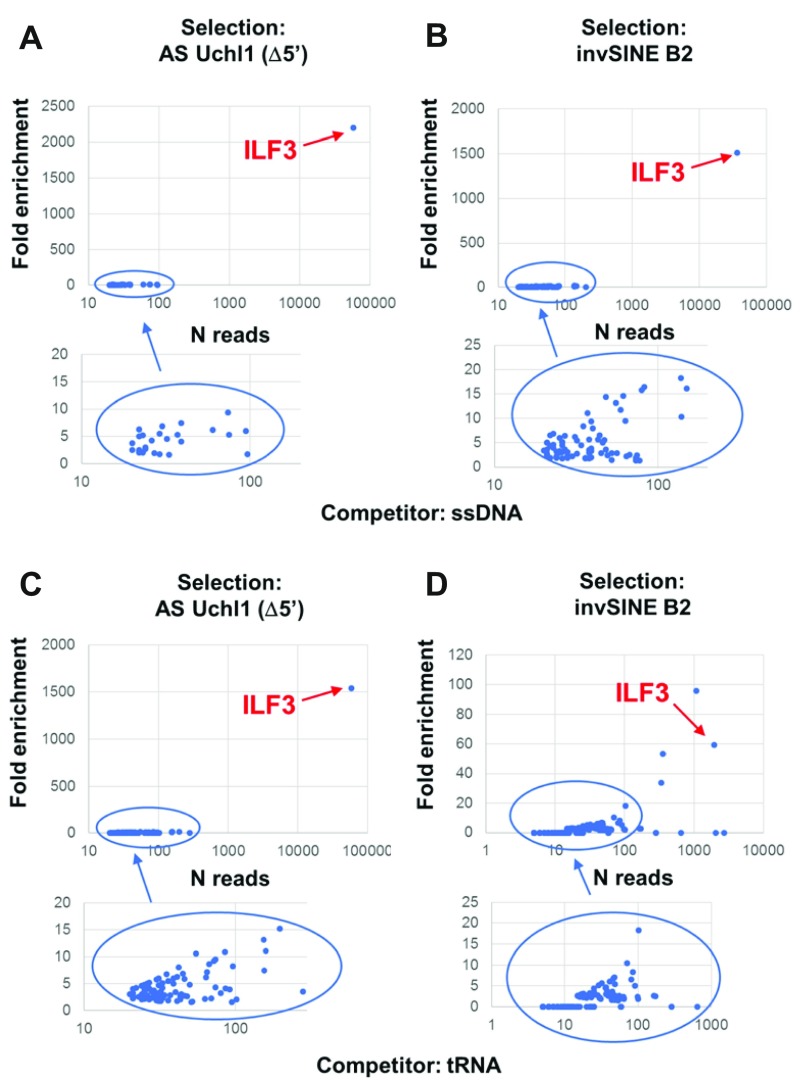
Summary of NGS analysis. Results from invSINEB2 and AS Uchl1 Δ5′selections are shown. *A*, *B*) Selections were carried out with ssDNA competitor. *C*, *D*) Selections were carried out with tRNA competitor. Enrichment analysis was performed by dividing the normalized number of reads (reads per million) in the selected libraries *vs.* the NS library. Genes were plotted on a dispersion graph showing the fold enrichment *vs.* the total number of reads. The blue circles correspond to enlarged areas in each chart.

We then performed a fold enrichment analysis to assess those genes whose ORFs were enriched after selection (37, 39). This analysis was carried out by comparing sequencing outputs of each selection with the NS library, the latter serving as a reference. Results are represented as 4 dispersion graphs showing the fold enrichment and the total number of reads for each represented gene (**Fig. 2**). In all selections, ILF3 [also known as nuclear factor (NF) 90 or 110 or DRBP76] scored as the top gene, having been enriched >1000-fold in 3 selections (Fig. 2*A*–*C*) and 60-fold in the fourth (Fig. 2*D*). Three additional genes were enriched exclusively as binders of the invSINEB2 sequence in the presence of tRNA as competitor: adaptor related protein complex 3 subunit δ1, DNAJ heat shock protein family member C7, and coiled-coil domain containing 124 (Fig. 2*D*). These were presenting features of parasitic clones that grow faster than the average phage library population, thus introducing biases in the selection process (57). Nevertheless, they were included in the validation pipeline, which did not confirm their interaction with invSINEB2 sequences in phage ELISA experiments, as expected (unpublished results).

**Figure 3:**
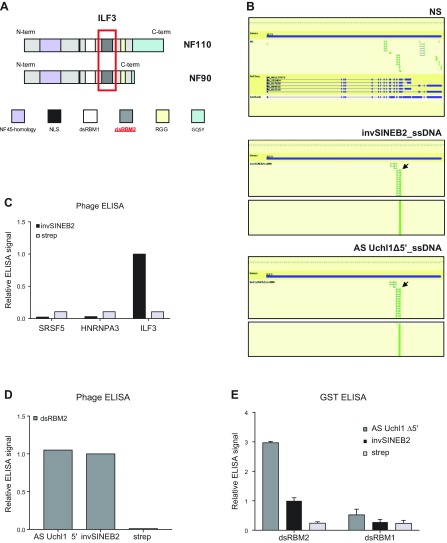
ILF3 is the dominant SINEUP-interacting ORF isolated by phage display selection. *A*) Schematic representation of ILF3 domains: NF45-homology domain, nuclear localization signal (NLS), dsRBMs 1 and 2, RGG motif, and GQSY domain. *B*) Reads alignment to *ILF3* gene showed specific enrichment of dsRBM2 (black arrows) in invSINEB2 library (middle) and AS Uchl1 Δ5′ library (bottom) but not in the NS library (top). Blue bars indicate the gene; green bars correspond to exons. *C*) Representative phage ELISA experiment of the binding of the invSINEB2 sequence to ILF3 and the RNA-recognition motif of negative controls (SRSF5 and hnRNPA3). *D*) Analysis by phage ELISA of the binding of dsRBM2 to AS Uchl1 Δ5′ and invSINEB2 RNA sequences. *E*) Analysis by GST ELISA of the binding specificity of ILF3 dsRBM1 and mouse-human dsRBM2 to AS Uchl1 Δ5′ and invSINEB2 RNA sequences. Domains were produced as GST fusion polypeptides. Strep, streptavidin. Data indicate means ± sd. Data are representative of *n* = 3 independent replicas.

ILF3 is a well-known dsRBP involved in many aspects of RNA biology. It presents 2 alternative forms, NF90 and NF110, generated by alternative splicing of the *ILF3* gene. They share common N-terminal and central sequences but display specific C-terminal regions (reviewed in ref. 58). They both contain 2 dsRBMs (referred to as dsRBM1 and dsRBM2) (**Fig. 3*A***). It is of note that the analysis of reads by NGS-Trex indicates a strong enrichment of ORFs overlapping the dsRBM2 of ILF3, as shown by the focus index increase from 0.21 (NS library) to >0.7 (selected libraries) (Fig. 3*B*). Importantly, because we screened a human library with a mouse RNA, it was necessary to verify that human and murine ILF3 proteins share 92% identity and 95% homology and that the dsRBM2 is identical in the 2 species (unpublished results), suggesting our data are representative of AS Uchl1–ILF3 interaction in the mouse.

We then focused our study on ILF3 to further investigate its binding to AS Uchl1. Firstly, the ILF3-dsRBM2 phage clones were rescued from the library by inverse PCR, using a primer pair targeting dsRBM2. Secondly, the binding to the invSINEB2 RNA was assessed by phage ELISA. As negative control, we used 2 phage clones expressing the RNA-recognition motifs of serine- and arginine-rich splicing factor 5 (SRSF5) and hnRNPA3, 2 known RBPs that were not enriched during the library selection. The phage expressing ILF3-dsRBM2 generated a strong signal on invSINEB2 compared with the negative control (wells coated with streptavidin alone), whereas the binding of SRSF5 and hnRNPA3 to invSINEB2 was negligible (Fig. 3*C*). We next validated the binding capacity of ILF3-dsRBM2 to bind to each of the 2 RNA baits in biopanning experiments. Results from phage ELISA experiments indicate that ILF3-dsRBM2 binds both AS Uchl1 ∆5′ and the invSINEB2 sequences to a similar extent (Fig. 3*D*). As further biochemical characterization, we compared the binding profiles of the 2 dsRBMs of ILF3. DsRBM1 and dsRBM2 were individually expressed as GST fusion proteins and assayed in ELISA for their binding to the RNA baits (Fig. 3*E*). The mouse-human dsRBM2-GST fusion protein showed strong binding to both baits, whereas binding to dsRBM1-GST was much weaker. It is notable that the binding to AS Uchl1 ∆5′ was characterized by a higher signal-to-noise ratio than to the invSINEB2 alone.

To further characterize the binding kinetics of the ILF3 mouse-human dsRBM2 to the invSINEB2, we used SPR. *In vitro* biotinylated invSINEB2 RNA was immobilized on a streptavidin-coated sensor chip analyzed on a Biacore T100, as described in Materials and Methods. The resulting sensorgram from invSINEB2-ILF3 interaction analysis did not totally adjust to a 1:1 binding model, as shown in Supplemental Fig. S2. However, data quality assessment indicated that kinetic parameters values were reliable for both interactions. Association rate (*K*_a_), dissociation rate (*k*_d_), and equilibrium dissociation constant or affinity constant *K*_d_ were calculated for the invSINEB2 RNA–ILF3 interaction after adjustment to this 1:1 binding model. A *K*_d_ of around 94.90 nM was calculated for this interaction, with association (*K*_a_) and dissociation rate (*k*_d_) constants equal to 3.84 × 10^4^ (M/s) and 3.64 × 10^3^ (s), respectively.

In summary, these results support a direct binding between the mouse invSINEB2 and the mouse-human ILF3 dsRBM2, which provides the specific domain mediating the interaction with AS Uchl1 baits *in vitro.*

### Upon ectopic expression, AS Uchl1 interacts with ILF3 in HEK 293T/17 cells, and the interaction requires the invSINEB2 sequence, the ED of mouse natural SINEUPs

**Figure 4:**
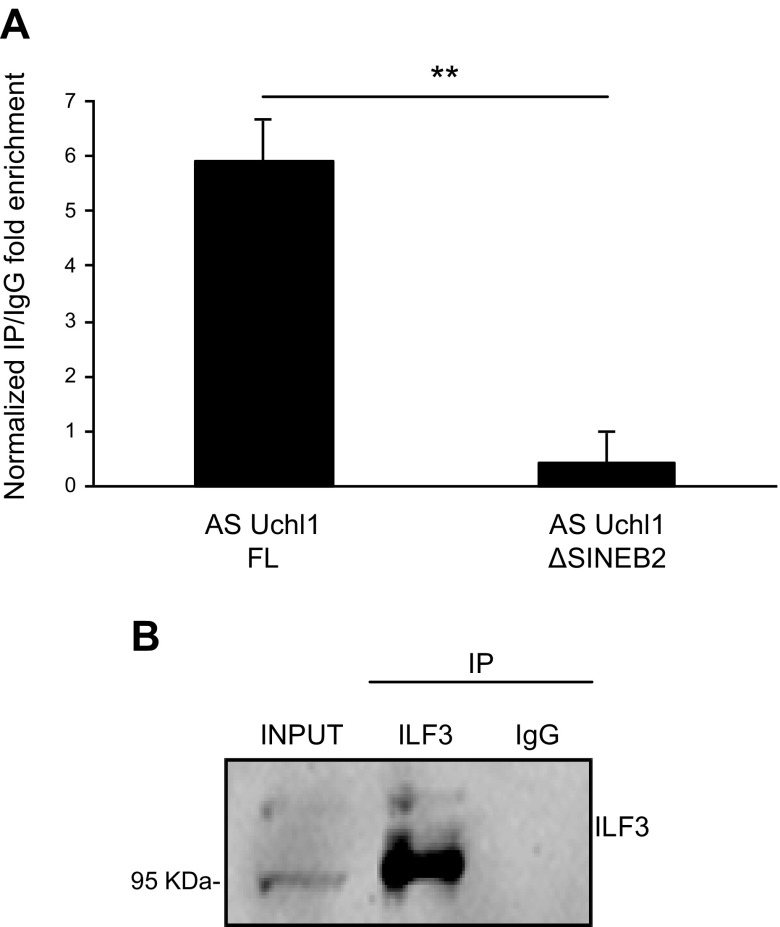
Validation of AS Uchl1-ILF3 interaction in HEK 293T/17 cells. *A*) Endogenous ILF3 was coimmunoprecipitated with ectopically expressed AS Uchl1 FL or AS Uchl1 ΔSINEB2 in HEK 293T/17. IgGs were used as control of immunoprecipitation (IP) specificity. FL or mutated AS Uchl1 enrichments in ILF3 IP fraction were quantified with real-time quantitative PCR and expressed as (2^Δ^*^Ct^*) × 100 ILF3 IP ÷ (2^Δ^*^Ct^*) × 100 IgG. Δ*C_t_* was calculated on input. RNA content in IP or IgG was normalized on UBC mRNA. *B*) ILF3 IP efficiency was monitored by Western blot performed with anti-DRBP76 antibody, recognizing both 90 and 110 kDa ILF3 isoforms. Data are representative of 3 independent experiments and indicate means ± sd. Differences of *P* < 0.05 were considered significant.

To validate and study AS Uchl1–ILF3 interaction in cells, we used the FL cDNA clone for AS Uchl1 (AS Uchl1-FL) (13) and carried out an RNA-IP assay on endogenous ILF3 in HEK 293T/17 cells. Following AS Uchl1-FL ectopic expression and cross-linking of RNA-protein complexes, endogenous ILF3 was immunoprecipitated with specific antibodies or control IgGs. The presence of target RNA in ILF3 immunoprecipitates *vs.* control was quantified by real-time quantitative PCR and normalized to the mRNA level of the housekeeping gene *UBC*, previously described in ref. 45 as noninteracting with ILF3. As shown in **Fig. 4*A***, AS Uchl1 was specifically enriched in ILF3 immunoprecipitates, confirming that ILF3 and AS Uchl1 interact in cells. Interestingly, Western blotting analysis showed a marked preference for the NF90 isoform (Fig. 4*B*). We also addressed the contribution of the embedded invSINEB2 to ILF3 binding. To this end, we used a deletion mutant of AS Uchl1 lacking the ED (AS Uchl1-ΔSINEB2) (13). As expected, the removal of the invSINEB2 abolished almost completely the binding of AS Uchl1 to ILF3 (Fig. 4*A*). Taken together, these results confirmed that the interaction between AS Uchl1 RNA and ILF3 occurs in cells upon AS Uchl1 ectopic expression and that the invSINEB2 is necessary for the binding.

### ILF3 interacts with FRAM, the ED of human SINEUP, *in vitro* and in HEK 293T/17 cells

**Figure 5:**
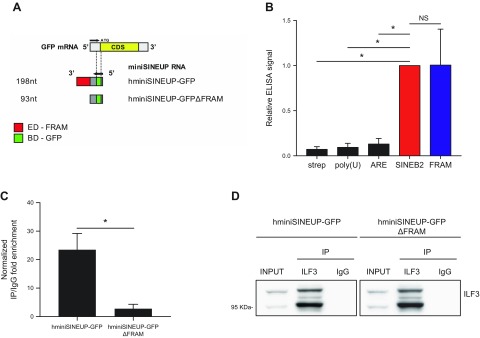
ILF3 binds the human FRAM *in vitro* and upon transfection of hminiSINEUP-GFP. *A*) Schematic representation of hminiSINEUP-GFP constructs. The overlapping region with sense GFP mRNA, representing the BD (green), spans 39 nt of GFP 5′UTR (gray). The FRAM is the ED (red) of hSINEUP R12A-AS1 (30). hminiSINEUP-GFPΔFRAM presents the BD but lacks the FRAM sequence. *B*) Analysis by phage ELISA of the binding of dsRBM2 to the human FRAM repeats RNA sequence. ELISA signals were normalized to the invSINEB2 of AS Uchl1 (SINEB2). As negative controls, bindings on streptavidin (strep) and 2 unrelated RNAs {polyuridine [poly(U)] and adenylate-uridylate–rich element (ARE)} were measured (*n* = 3). *C*) RNA-IP assay on endogenous ILF3 and ectopically expressed hminiSINEUP-GFP or hminiSINEUP-GFPΔFRAM in HEK 293T/17. IgGs were used as ILF3 immunoprecipitation (IP) specificity control. RNA enrichments in ILF3 IP fraction were quantified with real-time quantitative PCR and expressed as (2^Δ^*^Ct^*) × 100 ILF3 IP ÷ (2^Δ^*^Ct^*) × 100 IgG. Δ*C_t_* was calculated on input, and RNA content in IP or IgG was normalized on UBC mRNA. *D*) ILF3 IP efficiency was checked by Western blot performed with anti-DRBP76 (ILF3) antibody. Data are representative of 4 independent experiments and indicate means ± sd. NS, not significant. *P < 0.05.

Recently, R12A-AS1, NAT to human protein phosphatase 1 regulatory subunit 12A, has been shown to be the representative transcript for human natural SINEUPs. Its activity is mediated by an embedded FRAM acting as ED. FRAM, a human TE, supports SINEUP function when transferred to a chimeric AS RNA with BD that is AS to the mRNA of interest, including the 1 encoding for the green fluorescent protein (GFP) and called hminiSINEUP-GFP (30) (**Fig. 5*A***). Therefore, we investigated whether the FRAM element, the invSINEB2 human functional counterpart, is equally able to bind *in vitro* and upon ectopic expression to ILF3.

After transcribing the FRAM element *in vitro*, RNA was biotinylated and used in phage ELISA experiments as previously described. Results from 3 independent experiments are shown in Fig. 5*B*. After normalization to the signal for the invSINEB2 sequence, we could observe a similar binding to ILF3 for the human FRAM element.

An RNA-IP assay was then carried out on endogenous ILF3 following the ectopic expression of both synthetic hminiSINEUP-GFP having the FRAM element as ED and a deletion mutant lacking the ED (hminiSINEUP-GFP-ΔFRAM) in HEK 293T/17 cells. As shown in Fig. 5*C*, hminiSINEUP-GFP was specifically enriched in ILF3 immunoprecipitates, whereas the interaction of hminiSINEUP-GFP to ILF3 was completely abolished upon FRAM removal. ILF3 IP efficiency was checked by Western blot performed with anti-DRBP76 (ILF3) antibody (Figure 5*D*). Taken together, these results confirmed that the interaction between FRAM RNA and ILF3 occurs both *in vitro* and upon ectopic expression of hminiSINEUP in HEK 293T/17 cells.

### Bioinformatic analysis of eCLIP data for ILF3

**Figure 6:**
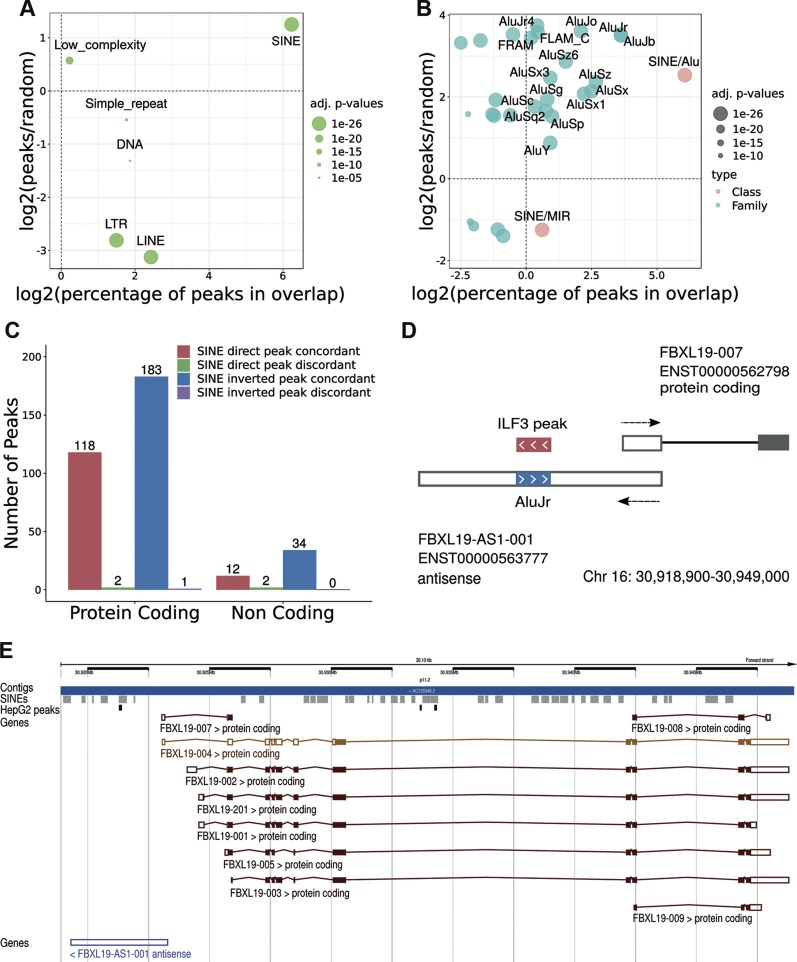
ILF3 binding analysis from ENCODE eCLIP data in human HepG2 cells. *A*) SINE class is the most frequent and enriched class of repeats in overlap with ILF3 peaks. *B*) The same analysis is carried out on SINE families (pink) and subfamilies (cyan). Enrichments are measured with respect to the genomic average resulting from randomizations and are appreciable on the *y* axes. Larger class, families, and subfamilies are on the right. Enrichments are shown as values above 0, whereas depletions are below. *C*) The numbers of peaks in SINE-containing exons are displayed according to different classes of coding and noncoding exons. *D*) Organization of the S/AS pair associated to the coding gene F-box and leucine-rich repeat protein 19 (FBXL19) whose AS contains an embedded inverted AluJr element bound by ILF3 from the eCLIP data. *E*) Genomic organization of the FBXL19 locus (Chr16:30,918,900–30,949,000) from the Ensembl genome browser. The track showing mapping of SINEs is in gray, whereas ILF3 peaks, loaded as custom tracks, are in black. LINE, long interspersed nuclear element; LTR, long terminal repeat; MIR, mammalian-wide interspersed repeat.

We next wondered whether ILF3 can bind other SINEs actively transcribed in the human genome. To this end, we took advantage of the publicly available UV CLIP data generated by ENCODE (40, 41). Focusing on ILF3, we could find experimental data from human HepG2 and K562 cell lines in physiologic conditions (42). We selected 14,224 total ILF3 peaks in HepG2 cells showing an enrichment value of *P* < 0.05 in both replicates. Considering the general mapping on the genome, more than 85% (12,125) of these peaks resulted in overlap with repeated elements. Randomization analysis demonstrated that SINEs are by far the most enriched TE (*P* < 1 × 10^−324^; **Fig. 6*A***). More than 88% (10,685) of repeat-overlapping peaks were in overlap with a SINE (Fig. 6*B*), of which 57% (6095) were on the reverse strand with respect to the SINE annotated strand. Most of the SINE-associated peaks overlapped with Alu elements, with the AluJ being the most significantly enriched subfamily (Fig. 6*B*). We observed also enrichments for many other SINE subfamilies, although to a much lower extent.

Analysis of the mapping with respect to the annotated genes revealed that about 98% (13,939) of the total peaks were in overlap with at least 1 genic region. Of these almost 96% (13,375) overlapped a coding gene, whereas 4% (564) overlapped a noncoding one. Most of genic overlaps, with respect to current annotation, were associated with introns. Indeed, only 7% of the coding genic peaks (951 peaks in 443 genes) and 16% of the noncoding ones (90 peaks in 36 genes) were exonic. The majority of exonic overlap was concordant with the strand of transcription (98% for coding and 93% for noncoding peaks).

When we then considered the association of ILF3 peaks overlapping SINEs embedded in annotated exons, we obtained a total of 304 peaks overlapping SINEs in exons from 172 coding genes and 48 peaks overlapping SINEs in 23 noncoding genes. In coding genes, 38% (118) of peaks overlapped with embedded direct SINEs, whereas 60% (183) overlapped with embedded inverted SINEs. In noncoding genes, 25% (12) of peaks overlapped embedded direct SINEs, whereas 70% (34) overlapped embedded inverted SINEs. The few remaining peaks overlapped on strands opposite to the annotated genes (Fig. 6*C*). Comparable results were obtained from the analysis of the K562 cell line (Supplemental Fig. S3). In Fig. 6*D* we show the genomic organization for the S/AS pair genes in the F-box and leucine-rich repeat protein 19 locus, where the AS contains an embedded inverted SINE with an ILF3-binding peak. The genomic organization of the FBXL19 locus (Chr16:30,918,900-30,949,000) from the Ensembl genome browser is shown in Figure 6*E*.

Using independent methodology, these results confirm ILF3 binding to SINEs embedded in coding and noncoding genes. In addition, the data demonstrate that ILF3 binds with a statistically significant preference for inverted elements.

### Embedded TEs modulate AS Uchl1 RNA nuclear localization, and its extent is moderately influenced by ILF3

**Figure 7:**
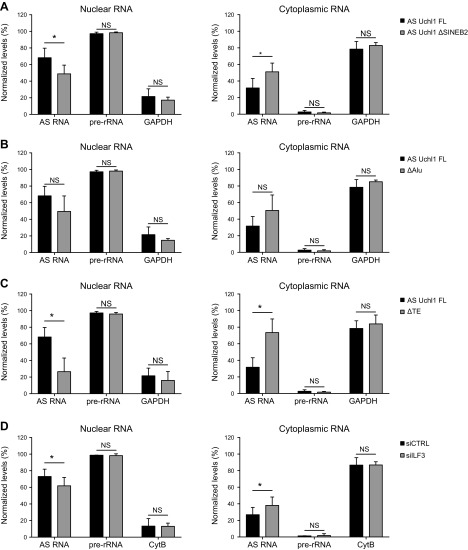
AS Uchl1 subcellular localization is affected by embedded TEs and ILF3. *A–C*) Following ectopic expression, subcellular distributions of AS Uchl1 ΔSINEB2 (*A*), ΔAlu (*B*), and ΔTE (*C*) were compared with that of AS Uchl1 FL (*n* = 4 independent experiments). *D*) Subcellular localization of ectopically expressed AS Uchl1 FL was evaluated in knocked-down cells for ILF3 (siILF3, Gy bars) (*n* = 3 independent experiments). Nucleocytoplasmic fractionation was performed, and RNA levels in nuclear (gray) and cytoplasmic (white) fractions were quantified by real-time quantitative PCR. Purity of cellular fractions was checked by monitoring levels of GAPDH, CytB, and pre-rRNA. Data are expressed as percentages of total RNA. Data indicate means ± sd. NS, not significant. *P < 0.05.

Because embedded TEs have been recently associated to nuclear localization of lncRNAs (59), we investigated whether the invSINEB2 is involved in AS Uchl1 RNA subcellular localization. To this end, we carried out cell fractionation from HEK 293T/17 cells transfected with AS Uchl1 FL or with AS Uchl1 ΔSINEB2. Levels in nuclear and cytoplasmic compartments were quantified by real-time quantitative PCR and expressed as relative percentages of total AS Uchl1 FL RNA. Purity of nuclear and cytoplasmic fractions was controlled by monitoring levels of GAPDH transcript and pre-rRNA, respectively. Real-time quantitative PCR data indicate that most of AS Uchl1 RNA (∼70%) was nuclear-retained. Interestingly, AS Uchl1 distribution was partially perturbed when the invSINEB2 was removed, with a 20% increase of cytoplasmic mutant RNA compared with the FL variant (**Fig. 7*A***).

Adjacent to the 3′ of the SINEB2 sequence, another TE, a partial Alu repeat, is present in the AS Uchl1 third exon (13). When an AS Uchl1 mutant lacking the Alu repeat [AS Uchl1 ΔAlu (13); Fig. 1*B*] was ectopically expressed, no statistically significant changes in subcellular localization were observed, although a trend similar to the deletion of the embedded invSINEB2 sequence was evident (Fig. 7*B*).

We finally investigated the effects of combined removal of the invSINEB2 and Alu elements (Fig. 1*B*) on RNA localization, proving that the absence of both TEs provoked a dramatic change of AS Uchl1 RNA distribution within cells, with 60–70% of total RNA accumulated in the cytoplasmic fraction (Fig. 7*C*).

To assess whether ILF3 may regulate AS Uchl1 RNA nuclear localization, we first carried out immunofluorescence analysis showing that endogenous ILF3 localizes in the nucleus of HEK 293T/17 cells, with no relevant signal in the cytoplasm (Supplemental Fig. S4*A*), suggesting that ILF3–AS Uchl1 RNA interaction is likely to occur in the nucleus.

To test whether ILF3 was required for AS Uchl1 nuclear entrapment, we ectopically expressed AS Uchl1 FL in ILF3-silenced HEK293/17 cells and checked its subcellular localization upon nucleocytoplasmic fractionation (Fig. 7*D*). In conditions of highly efficient ILF3 knockdown (Supplemental Fig. S4*B*), data showed a 10–20% increase of AS Uchl1 FL in the cytoplasmic fraction phenocopying the effects of invSINEB2 removal. RNA distribution was confirmed with 2 different control transcripts.

## DISCUSSION

The diversity of lncRNAs’ activity and function mainly depends on their modular architecture and their physical interactions with proteins. To understand the basic rules of this molecular network, we need to identify RNA sequences able to independently fold into functional secondary structures and the proteins that interact with them in a regulated fashion.

We and others have proposed that embedded TEs may represent independent structural modules with specific roles in lncRNAs, whose function is exerted through RNA-protein interactions (14–16). We have previously shown that in the murine AS Uchl1 lncRNA, an embedded invSINEB2 acts as an ED that is required to increase translation of the target mRNA. Here we aimed to identify proteins that interact with this TE and anticipated that the data generated would help to reveal aspects of the molecular mechanisms governing the subcellular localization and activity of SINEUPs. To this end, we took advantage of the RIDome technology, recently proposed for investigating RNA-RBP interactions (36). By combining *in vitro* phage display selection with NGS, this method provides an unbiased, high-throughput approach to study RNA-protein interactions. ORF phage libraries can faithfully represent whole proteomes or domainomes of cells, with the advantage of coupling phenotype to genotype identification. Furthermore, when ORF domain libraries are employed, the specific domains involved in bait-binding can be identified. Because this approach allows multiple screenings to be run on selected transcripts domains, we carried out 4 parallel selections, using 2 different RNA baits and 2 competitors to ensure reproducibility and robustness of our selection procedure. The RNA baits were *1*) the invSINEB2 of AS Uchl1, where it exerts its ED function common to all mouse natural SINEUPs, and *2*) AS Uchl1 Δ5′, an RNA lacking the BD but including the ED in an embedded format. This construct provides the backbone on which synthetic SINEUPs are built (13, 29). Both RNA baits contain the invSINEB2 sequence. This choice was motivated by the possibility that the embedded SINEB2 may fold differently than the solitary element, giving rise to secondary structures that do not correspond to those formed in the natural lncRNA. We avoided the use of FL AS Uchl1 because its function should require the formation of a dsRNA sequence that would have obliged the use of paired S/AS transcripts as baits, a condition difficult to reproduce in an *in vitro* assay. However, future screenings should also investigate the repertory of interactors of FL AS Uchl1.

By using this approach, after several rounds of selection, ILF3 was the most enriched gene in the data set. Although its ORFs were enriched >1000-fold in 3 selections, the extent of enrichment was substantially lower in the screening for binders of the invSINEB2 sequence in the presence of tRNA as competitor. The cause of this difference remains unclear. ELISA experiments confirmed the interaction between the invSINEB2 and ILF3 *in vitro*. ILF3 is a ubiquitously expressed dsRBP. Initially identified as a transcription factor in the IL-2 promoter-binding complex (60, 61), ILF3 was later found to be involved in diverse processes besides transcription, including splicing and translation, and more generally in RNA metabolism, including transport, localization, and stability (58). Protein isoforms are generated by a combination of alternative splicing and differential polyadenylation events, with the most abundant splicing variants known as NF90 and NF110, of 90 and 110 kDa, respectively. These proteins differ by an additional ∼200 aa in the NF110 C terminus. RNA-binding capability mainly relies on the 2 dsRBMs, referred to as dsRBM1 and dsRBM2 (62). Our data suggest a direct binding between the dsRBM2 of ILF3 and the lncRNA AS Uchl1. Alignments of ILF3 reads relative to both invSINEB2 and AS Uchl1 Δ5′ selection outputs showed exclusive mapping on dsRBM2, whereas sequencing of the NS library confirmed that such enrichment was exclusively maintained after stringent selection. The binding of ILF3-dsRBM2 to the invSINEB2 was also validated experimentally *in vitro* by ELISA. Interestingly, interaction of dsRBM2 with AS Uchl1 Δ5′ was characterized by a better signal-to-noise ratio compared with the invSINEB2 alone. We speculate that it might be linked to a more appropriate RNA folding, when present in an embedded format, or to a role of the adjacent Alu sequence. Importantly, no binding was observed between dsRBM1 and any portion of AS Uchl1 sequence *in vitro*.

By SPR analysis we measured invSINEB2-ILF3 binding kinetics *in vitro*. A *K*_d_ of around 0.1 μM was calculated for this interaction, although the *K*_a_ and *k*_d_ constants were slightly different. This value is in agreement with recently published data, where a *K*_d_ of 160 nM has been measured for the interaction between ILF3 (NF90) and a dsRNA (63). It should be noted, however, that the affinity of ILF3 for a dsRNA is strongly dependent on the nature of the dsRNA substrate, and it is considerably modulated by complex formation with NF45 with binding affinities reported in the range 0.1–2.5 μM (63–65). The fact that the invSINEB2 RNA–ILF3 complex does not completely adjust to a 1:1 model could be due to a range of factors, like multiple binding sites on the ligand (RNA), a conformational change after a first contact between the 2 molecules, or a heterogeneous sample preparation, among others. Further experiments are required to elucidate these points.

ILF3–AS Uchl1 interaction and its reliance on the embedded TE were then demonstrated by experiments in HEK 293T/17 cells upon ectopic expression of the lncRNA transcript. A reproducible enrichment of AS Uchl1 RNA was revealed in endogenous ILF3 immunoprecipitates, which was substantially reduced on deletion of invSINEB2. Although AS Uchl1 is a mouse transcript and ILF3 synthesized from the phage library and in HEK 293T/17 cells is of the human type, we considered these results also representative of the interaction in mouse given the 92% identity and 95% homology between human and mouse ILF3 protein sequences and the 100% conservation of dsRBM2, the invSINEB2 BD of ILF3. Future ILF3 immunoprecipitation experiments should be carried out in mouse cells to experimentally prove the interaction of the FL rodent protein with AS Uchl1. Nevertheless, because SINEB2 sequences are not present in the human transcriptome, we also demonstrated that ILF3 was able to bind FRAM, the ED in human natural SINEUPs. Although SINEB2 and FRAM do not present extensive homology at the primary sequence and there is no clear consensus sequence for ILF3 binding, our results suggest that they form conserved secondary structures that are able to bind common interacting partners. This result is relevant under the hypothesis that embedded TEs can act as convergent functional domains.

We then asked whether ILF3-FRAM interaction is a representative example of a larger pattern of ILF3 binding to SINE sequences in the mammalian transcriptome. To this end we took advantage of ENCODE eCLIP data for ILF3 in 2 human cell lines. In general, ILF3 binding to SINEs was extremely strong, proving a highly significant and specific preference of ILF3 for transcribed fragments containing these elements. The presence of multiple additional ILF3 binding interactions with introns, coding exons, noncoding non-AS exons, and SINEs on the transcribed strand probably reflects the extensive functional diversity of the ILF3 gene in addition to an incomplete annotation of the transcriptome. It remains to be determined whether different levels of enrichment for Alu families reflect distinctive RNA secondary structures and protein binding profiles, opening up an interesting topic of investigation on the diversity of functional roles of embedded Alus in lncRNAs.

Earlier data have demonstrated that TEs of the SINEs and Alu families are involved in the RNA association with nuclear protein complexes, which subsequently control RNA export and cytoplasmic availability (66, 67). More recently, SINEs have been shown to drive nuclear retention of lncRNAs (59). Because AS Uchl1 is enriched in the nucleus of neurons *in vitro* and *in vivo* (13), we monitored AS Uchl1 distribution upon ectopic expression in HEK 293T/17 cells, proving that it accumulates in the nucleus as well. Importantly, a moderate but significant cytoplasmic accumulation occurred upon removal of the invSINEB2.

Recently, inverted repeat Alu elements embedded in long intergenic noncoding RNA-p21 have been shown to fold into specific structures required for RNA nuclear localization. Mutations disrupting such secondary structures resulted in altered long intergenic noncoding RNA-p21 distribution (66, 68). According to this model, tandem invSINEB2 and Alu elements would provide heterodimeric repeats (69) dictating AS Uchl1 nuclear localization. We thus hypothesized that a partial Alu sequence present at the 3′ of the SINEB2 may participate in AS Uchl1 nuclear retention. Alu’s deletion did affect AS Uchl1 subcellular localization, although its effect did not reach statistical significance, probably because of the large variation between experimental replicas. However, combined removal of the invSINEB2 and Alu elements significantly altered AS Uchl1 RNA distribution with about 70% shuffling to the cytoplasmic compartment.

As previously shown for other cellular systems (70–72), ILF3 is almost exclusively localized in cell nuclei of HEK 293T/17 cells. Therefore, we investigated whether the ILF3–AS Uchl1 RNA interaction may be involved in AS Uchl1 nuclear retention. When ILF3 was silenced with siRNAs, a reproducible and significant 10–20% increase in cytoplasmic content of AS Uchl1 was observed, phenocopying the removal of the invSINEB2. Several reasons may account for the moderate influence of ILF3 removal on AS Uchl1 nuclear restriction. Firstly, ILF3 has multiple functions exerted through a complex pattern of protein interactions. We may envision that other partners are mediating ILF3 influence of RNA nuclear localization. Secondly, we are ectopically expressing a cDNA clone, which may result in loss of the potential regulatory interplay between splicing and nuclear retention. In addition, recent works suggest that regulated chemical modifications play a crucial role in RNA nuclear export (73). At present, nothing is known about AS Uchl1 RNA post-transcriptional modifications and whether they are accurately reproduced in an ectopically expressed RNA.

Therefore, the structural requirements for the embedded heterodimeric repeat composed of the invSINEB2 and the truncated Alu remain to be defined, along with the identity of additional protein partners and the details of their interactions with ILF3. In addition, future studies will investigate the biologic significance of a 20% increase in cytoplasmic AS Uchl1 RNA, including its effect on the ability to regulate endogenous protein levels of its RNA sense target.

In summary, through the identification of ILF3 as a binding partner of mouse invSINEB2 and human FRAM embedded in SINEUP lncRNAs, we provide strong evidence that TEs act as functional modules in lncRNAs. By detailed bioinformatic analysis of eCLIP data, we showed that ILF3 binding sequences are highly enriched for SINEs embedded in human transcripts. We then demonstrated that nuclear localization of AS Uchl RNA depends on embedded TEs and is moderately influenced by ILF3. This work paves the way for further studies on the biologic role of interactions between ILF3 and embedded TEs in lncRNA dynamics and function.

## Supplementary Material

This article includes supplemental data. Please visit *http://www.fasebj.org* to obtain this information.

## Abbreviations

Alu: *Arthrobacter luteus*
AS: antisense
BD: binding domain
BSA: bovine serum albumin
CLIP: cross-linking immunoprecipitation
CytB: cytochrome B
dsRBM: dsRNA-binding motif
dsRBP: double-stranded RBP
dsRNA: double-stranded RNA
eCLIP: enhanced CLIP
ED: effector domain
ENCODE: Encyclopedia of DNA Elements
FBS: fetal bovine serum
FL: full length
FRAM: free right Alu monomer
GAPDH: glyceraldehyde 3-phosphate dehydrogenase
GFP: green fluorescent protein
GST: glutathione *S*-transferase
HEK: human embryonic kidney
hnRNP: heterogeneous ribonucleoprotein particle
HRP: horseradish peroxidase
ILF3: IL enhancer-binding factor 3
invSINEB2: inverted SINE of subfamily B2
lncRNA: long noncoding RNA
NAT: natural AS transcript
NF: nuclear factor
NGS: next-generation sequencing
NGS-Trex: NGS Transcriptome Profile Explorer
NS: nonselected
ORF: open reading frame
pre-rRNA: precursor rRNA
RBP: RNA-binding protein
RIDome: RNA-interacting domainome
RNA-IP: RNA immunoprecipitation
S/AS: sense-AS
SINE: short interspersed nuclear element
siRNA: small interfering RNA
SPR: surface plasmon resonance
SRSF5: serine- and arginine-rich splicing factor 5
ssDNA: single-stranded DNA
TE: transposable element
TENT buffer: 10 mM Tris HCl pH 8.0, 1 mM EDTA, 250 mM NaCl, 0.5% Triton X-100
UBC: ubiquitin C
Uchl1: ubiquitin C-terminal hydrolase L1

## References

[1] Neme R., Tautz D. (2016) Fast turnover of genome transcription across evolutionary time exposes entire non-coding DNA to *de novo* gene emergence. eLife 5, e09977 26836309 10.7554/eLife.09977 PMC4829534

[2] Derrien T., Johnson R., Bussotti G., Tanzer A., Djebali S., Tilgner H., Guernec G., Martin D., Merkel A., Knowles D. G., Lagarde J., Veeravalli L., Ruan X., Ruan Y., Lassmann T., Carninci P., Brown J. B., Lipovich L., Gonzalez J. M., Thomas M., Davis C. A., Shiekhattar R., Gingeras T. R., Hubbard T. J., Notredame C., Harrow J., Guigó R. (2012) The GENCODE v7 catalog of human long noncoding RNAs: analysis of their gene structure, evolution, and expression. Genome Res. 22, 1775–1789 22955988 10.1101/gr.132159.111 PMC3431493

[3] Hon C. C., Ramilowski J. A., Harshbarger J., Bertin N., Rackham O. J., Gough J., Denisenko E., Schmeier S., Poulsen T. M., Severin J., Lizio M., Kawaji H., Kasukawa T., Itoh M., Burroughs A. M., Noma S., Djebali S., Alam T., Medvedeva Y. A., Testa A. C., Lipovich L., Yip C. W., Abugessaisa I., Mendez M., Hasegawa A., Tang D., Lassmann T., Heutink P., Babina M., Wells C. A., Kojima S., Nakamura Y., Suzuki H., Daub C. O., de Hoon M. J., Arner E., Hayashizaki Y., Carninci P., Forrest A. R. (2017) An atlas of human long non-coding RNAs with accurate 5′ ends. Nature 543, 199–204 28241135 10.1038/nature21374 PMC6857182

[4] Forrest A. R., Kawaji H., Rehli M., Baillie J. K., de Hoon M. J., Haberle V., Lassmann T., Kulakovskiy I. V., Lizio M., Itoh M., Andersson R., Mungall C. J., Meehan T. F., Schmeier S., Bertin N., Jørgensen M., Dimont E., Arner E., Schmidl C., Schaefer U., Medvedeva Y. A., Plessy C., Vitezic M., Severin J., Semple C., Ishizu Y., Young R. S., Francescatto M., Alam I., Albanese D., Altschuler G. M., Arakawa T., Archer J. A., Arner P., Babina M., Rennie S., Balwierz P. J., Beckhouse A. G., Pradhan-Bhatt S., Blake J. A., Blumenthal A., Bodega B., Bonetti A., Briggs J., Brombacher F., Burroughs A. M., Califano A., Cannistraci C. V., Carbajo D., Chen Y., Chierici M., Ciani Y., Clevers H. C., Dalla E., Davis C. A., Detmar M., Diehl A. D., Dohi T., Drabløs F., Edge A. S., Edinger M., Ekwall K., Endoh M., Enomoto H., Fagiolini M., Fairbairn L., Fang H., Farach-Carson M. C., Faulkner G. J., Favorov A. V., Fisher M. E., Frith M. C., Fujita R., Fukuda S., Furlanello C., Furino M., Furusawa J., Geijtenbeek T. B., Gibson A. P., Gingeras T., Goldowitz D., Gough J., Guhl S., Guler R., Gustincich S., Ha T. J., Hamaguchi M., Hara M., Harbers M., Harshbarger J., Hasegawa A., Hasegawa Y., Hashimoto T., Herlyn M., Hitchens K. J., Ho Sui S. J., Hofmann O. M., Hoof I., Hori F., Huminiecki L., Iida K., Ikawa T., Jankovic B. R., Jia H., Joshi A., Jurman G., Kaczkowski B., Kai C., Kaida K., Kaiho A., Kajiyama K., Kanamori-Katayama M., Kasianov A. S., Kasukawa T., Katayama S., Kato S., Kawaguchi S., Kawamoto H., Kawamura Y. I., Kawashima T., Kempfle J. S., Kenna T. J., Kere J., Khachigian L. M., Kitamura T., Klinken S. P., Knox A. J., Kojima M., Kojima S., Kondo N., Koseki H., Koyasu S., Krampitz S., Kubosaki A., Kwon A. T., Laros J. F., Lee W., Lennartsson A., Li K., Lilje B., Lipovich L., Mackay-Sim A., Manabe R., Mar J. C., Marchand B., Mathelier A., Mejhert N., Meynert A., Mizuno Y., de Lima Morais D. A., Morikawa H., Morimoto M., Moro K., Motakis E., Motohashi H., Mummery C. L., Murata M., Nagao-Sato S., Nakachi Y., Nakahara F., Nakamura T., Nakamura Y., Nakazato K., van Nimwegen E., Ninomiya N., Nishiyori H., Noma S., Noma S., Noazaki T., Ogishima S., Ohkura N., Ohimiya H., Ohno H., Ohshima M., Okada-Hatakeyama M., Okazaki Y., Orlando V., Ovchinnikov D. A., Pain A., Passier R., Patrikakis M., Persson H., Piazza S., Prendergast J. G., Rackham O. J., Ramilowski J. A., Rashid M., Ravasi T., Rizzu P., Roncador M., Roy S., Rye M. B., Saijyo E., Sajantila A., Saka A., Sakaguchi S., Sakai M., Sato H., Savvi S., Saxena A., Schneider C., Schultes E. A., Schulze-Tanzil G. G., Schwegmann A., Sengstag T., Sheng G., Shimoji H., Shimoni Y., Shin J. W., Simon C., Sugiyama D., Sugiyama T., Suzuki M., Suzuki N., Swoboda R. K., ’t Hoen P. A., Tagami M., Takahashi N., Takai J., Tanaka H., Tatsukawa H., Tatum Z., Thompson M., Toyodo H., Toyoda T., Valen E., van de Wetering M., van den Berg L. M., Verado R., Vijayan D., Vorontsov I. E., Wasserman W. W., Watanabe S., Wells C. A., Winteringham L. N., Wolvetang E., Wood E. J., Yamaguchi Y., Yamamoto M., Yoneda M., Yonekura Y., Yoshida S., Zabierowski S. E., Zhang P. G., Zhao X., Zucchelli S., Summers K. M., Suzuki H., Daub C. O., Kawai J., Heutink P., Hide W., Freeman T. C., Lenhard B., Bajic V. B., Taylor M. S., Makeev V. J., Sandelin A., Hume D. A., Carninci P., Hayashizaki Y.; FANTOM Consortium and the RIKEN PMI and CLST (DGT) (2014) A promoter-level mammalian expression atlas. Nature 507, 462–470 24670764 10.1038/nature13182 PMC4529748

[5] Iyer M. K., Niknafs Y. S., Malik R., Singhal U., Sahu A., Hosono Y., Barrette T. R., Prensner J. R., Evans J. R., Zhao S., Poliakov A., Cao X., Dhanasekaran S. M., Wu Y. M., Robinson D. R., Beer D. G., Feng F. Y., Iyer H. K., Chinnaiyan A. M. (2015) The landscape of long noncoding RNAs in the human transcriptome. Nat. Genet. 47, 199–208 25599403 10.1038/ng.3192 PMC4417758

[6] Volders P. J., Verheggen K., Menschaert G., Vandepoele K., Martens L., Vandesompele J., Mestdagh P. (2015) An update on LNCipedia: a database for annotated human lncRNA sequences. Nucleic Acids Res. 43, 4363–4364 25829178 10.1093/nar/gkv295 PMC4417186

[7] Morris K. V., Mattick J. S. (2014) The rise of regulatory RNA. Nat. Rev. Genet. 15, 423–437 24776770 10.1038/nrg3722 PMC4314111

[8] Marín-Béjar O., Huarte M. (2015) Long noncoding RNAs: from identification to functions and mechanisms. Adv. Genomics Genet. 5, 257–274

[9] Engström P. G., Suzuki H., Ninomiya N., Akalin A., Sessa L., Lavorgna G., Brozzi A., Luzi L., Tan S. L., Yang L., Kunarso G., Ng E. L., Batalov S., Wahlestedt C., Kai C., Kawai J., Carninci P., Hayashizaki Y., Wells C., Bajic V. B., Orlando V., Reid J. F., Lenhard B., Lipovich L. (2006) Complex loci in human and mouse genomes. PLoS Genet. 2, e47 16683030 10.1371/journal.pgen.0020047 PMC1449890

[10] Faghihi M. A., Wahlestedt C. (2009) Regulatory roles of natural antisense transcripts. Nat. Rev. Mol. Cell Biol. 10, 637–643 19638999 10.1038/nrm2738 PMC2850559

[11] Wight M., Werner A. (2013) The functions of natural antisense transcripts. Essays Biochem. 54, 91–101 23829529 10.1042/bse0540091 PMC4284957

[12] Guttman M., Rinn J. L. (2012) Modular regulatory principles of large non-coding RNAs. Nature 482, 339–346 22337053 10.1038/nature10887 PMC4197003

[13] Carrieri C., Cimatti L., Biagioli M., Beugnet A., Zucchelli S., Fedele S., Pesce E., Ferrer I., Collavin L., Santoro C., Forrest A. R., Carninci P., Biffo S., Stupka E., Gustincich S. (2012) Long non-coding antisense RNA controls Uchl1 translation through an embedded SINEB2 repeat. Nature 491, 454–457 23064229 10.1038/nature11508

[14] Johnson R., Guigó R. (2014) The RIDL hypothesis: transposable elements as functional domains of long noncoding RNAs. RNA 20, 959–976 24850885 10.1261/rna.044560.114 PMC4114693

[15] Kapusta A., Feschotte C. (2014) Volatile evolution of long noncoding RNA repertoires: mechanisms and biological implications. Trends Genet. 30, 439–452 25218058 10.1016/j.tig.2014.08.004 PMC4464757

[16] Zucchelli S., Cotella D., Takahashi H., Carrieri C., Cimatti L., Fasolo F., Jones M. H., Sblattero D., Sanges R., Santoro C., Persichetti F., Carninci P., Gustincich S. (2015) SINEUPs: a new class of natural and synthetic antisense long non-coding RNAs that activate translation. RNA Biol. 12, 771–779 26259533 10.1080/15476286.2015.1060395 PMC4615742

[17] Cordaux R., Batzer M. A. (2009) The impact of retrotransposons on human genome evolution. Nat. Rev. Genet. 10, 691–703 19763152 10.1038/nrg2640 PMC2884099

[18] Kapusta A., Kronenberg Z., Lynch V. J., Zhuo X., Ramsay L., Bourque G., Yandell M., Feschotte C. (2013) Transposable elements are major contributors to the origin, diversification, and regulation of vertebrate long noncoding RNAs. PLoS Genet. 9, e1003470 23637635 10.1371/journal.pgen.1003470 PMC3636048

[19] Kelley D., Rinn J. (2012) Transposable elements reveal a stem cell-specific class of long noncoding RNAs. Genome Biol. 13, R107 23181609 10.1186/gb-2012-13-11-r107 PMC3580499

[20] Holdt L. M., Hoffmann S., Sass K., Langenberger D., Scholz M., Krohn K., Finstermeier K., Stahringer A., Wilfert W., Beutner F., Gielen S., Schuler G., Gäbel G., Bergert H., Bechmann I., Stadler P. F., Thiery J., Teupser D. (2013) Alu elements in ANRIL non-coding RNA at chromosome 9p21 modulate atherogenic cell functions through trans-regulation of gene networks. PLoS Genet. 9, e1003588 23861667 10.1371/journal.pgen.1003588 PMC3701717

[21] Gong C., Maquat L. E. (2011) lncRNAs transactivate STAU1-mediated mRNA decay by duplexing with 3′ UTRs via Alu elements. Nature 470, 284–288 21307942 10.1038/nature09701 PMC3073508

[22] Ricci E. P., Kucukural A., Cenik C., Mercier B. C., Singh G., Heyer E. E., Ashar-Patel A., Peng L., Moore M. J. (2014) Staufen1 senses overall transcript secondary structure to regulate translation. Nat. Struct. Mol. Biol. 21, 26–35 24336223 10.1038/nsmb.2739 PMC4605437

[23] Zarnack K., König J., Tajnik M., Martincorena I., Eustermann S., Stévant I., Reyes A., Anders S., Luscombe N. M., Ule J. (2013) Direct competition between hnRNP C and U2AF65 protects the transcriptome from the exonization of Alu elements. Cell 152, 453–466 23374342 10.1016/j.cell.2012.12.023 PMC3629564

[24] Tollervey J. R., Curk T., Rogelj B., Briese M., Cereda M., Kayikci M., König J., Hortobágyi T., Nishimura A. L., Zupunski V., Patani R., Chandran S., Rot G., Zupan B., Shaw C. E., Ule J. (2011) Characterizing the RNA targets and position-dependent splicing regulation by TDP-43. Nat. Neurosci. 14, 452–458 21358640 10.1038/nn.2778 PMC3108889

[25] Kelley D. R., Hendrickson D. G., Tenen D., Rinn J. L. (2014) Transposable elements modulate human RNA abundance and splicing via specific RNA-protein interactions. Genome Biol. 15, 537 25572935 10.1186/s13059-014-0537-5 PMC4272801

[26] Katayama S., Tomaru Y., Kasukawa T., Waki K., Nakanishi M., Nakamura M., Nishida H., Yap C. C., Suzuki M., Kawai J., Suzuki H., Carninci P., Hayashizaki Y., Wells C., Frith M., Ravasi T., Pang K. C., Hallinan J., Mattick J., Hume D. A., Lipovich L., Batalov S., Engström P. G., Mizuno Y., Faghihi M. A., Sandelin A., Chalk A. M., Mottagui-Tabar S., Liang Z., Lenhard B., Wahlestedt C.; RIKEN Genome Exploration Research Group; Genome Science Group (Genome Network Project Core Group); FANTOM Consortium (2005) Antisense transcription in the mammalian transcriptome. Science 309, 1564–1566 16141073 10.1126/science.1112009

[27] Pelechano V., Steinmetz L. M. (2013) Gene regulation by antisense transcription. Nat. Rev. Genet. 14, 880–893 24217315 10.1038/nrg3594

[28] Zucchelli S., Fedele S., Vatta P., Calligaris R., Heutink P., Rizzu P., Itoh M., Persichetti F., Santoro C., Kawaji H., Lassmann T., Hayashizaki Y., Carninci P., Forrest A. R. R., Gustincich S.; FANTOM Consortium (2019) Antisense transcription in loci associated to hereditary neurodegenerative diseases. Mol. Neurobiol. 56, 5392–5415 30610612 10.1007/s12035-018-1465-2 PMC6614138

[29] Zucchelli S., Fasolo F., Russo R., Cimatti L., Patrucco L., Takahashi H., Jones M. H., Santoro C., Sblattero D., Cotella D., Persichetti F., Carninci P., Gustincich S. (2015) SINEUPs are modular antisense long non-coding RNAs that increase synthesis of target proteins in cells. Front. Cell. Neurosci. 9, 174 26029048 10.3389/fncel.2015.00174 PMC4429562

[30] Schein A., Zucchelli S., Kauppinen S., Gustincich S., Carninci P. (2016) Identification of antisense long noncoding RNAs that function as SINEUPs in human cells. Sci. Rep. 6, 33605 27646849 10.1038/srep33605 PMC5028707

[31] Gustincich S., Zucchelli S., Mallamaci A. (2017) The Yin and Yang of nucleic acid-based therapy in the brain. Prog. Neurobiol. 155, 194–211 27887908 10.1016/j.pneurobio.2016.11.001

[32] Indrieri A., Grimaldi C., Zucchelli S., Tammaro R., Gustincich S., Franco B. (2016) Synthetic long non-coding RNAs [SINEUPs] rescue defective gene expression *in vivo*. Sci. Rep. 6, 27315 27265476 10.1038/srep27315 PMC4893607

[33] Patrucco L., Chiesa A., Soluri M. F., Fasolo F., Takahashi H., Carninci P., Zucchelli S., Santoro C., Gustincich S., Sblattero D., Cotella D. (2015) Engineering mammalian cell factories with SINEUP noncoding RNAs to improve translation of secreted proteins. Gene 569, 287–293 26045368 10.1016/j.gene.2015.05.070

[34] Podbevšek P., Fasolo F., Bon C., Cimatti L., Reißer S., Carninci P., Bussi G., Zucchelli S., Plavec J., Gustincich S. (2018) Structural determinants of the SINE B2 element embedded in the long non-coding RNA activator of translation AS Uchl1. Sci. Rep. 8, 3189 29453387 10.1038/s41598-017-14908-6 PMC5816658

[35] Sanchez de Groot N., Armaos A., Graña-Montes R., Alriquet M., Calloni G., Vabulas R. M., Tartaglia G. G. (2019) RNA structure drives interaction with proteins. Nat. Commun. 10, 3246 31324771 10.1038/s41467-019-10923-5 PMC6642211

[36] Patrucco L., Peano C., Chiesa A., Guida F., Luisi I., Boria I., Mignone F., De Bellis G., Zucchelli S., Gustincich S., Santoro C., Sblattero D., Cotella D. (2015) Identification of novel proteins binding the AU-rich element of α-prothymosin mRNA through the selection of open reading frames (RIDome). RNA Biol. 12, 1289–1300 26512911 10.1080/15476286.2015.1107702 PMC4829324

[37] Di Niro R., Sulic A. M., Mignone F., D’Angelo S., Bordoni R., Iacono M., Marzari R., Gaiotto T., Lavric M., Bradbury A. R., Biancone L., Zevin-Sonkin D., De Bellis G., Santoro C., Sblattero D. (2010) Rapid interactome profiling by massive sequencing. Nucleic Acids Res. 38, e110 20144949 10.1093/nar/gkq052 PMC2875021

[38] Deantonio C., Cotella D., Macor P., Santoro C., Sblattero D. (2014) Phage display technology for human monoclonal antibodies. Methods Mol. Biol. 1060, 277–295 24037846 10.1007/978-1-62703-586-6_14

[39] Boria I., Boatti L., Pesole G., Mignone F. (2013) NGS-Trex: next generation sequencing transcriptome profile explorer. BMC Bioinformatics 14 (Suppl 7), S10 10.1186/1471-2105-14-S7-S10 PMC3633008 23815181

[40] ENCODE Project Consortium (2012) An integrated encyclopedia of DNA elements in the human genome. Nature 489, 57–74 22955616 10.1038/nature11247 PMC3439153

[41] Sloan C. A., Chan E. T., Davidson J. M., Malladi V. S., Strattan J. S., Hitz B. C., Gabdank I., Narayanan A. K., Ho M., Lee B. T., Rowe L. D., Dreszer T. R., Roe G., Podduturi N. R., Tanaka F., Hong E. L., Cherry J. M. (2016) ENCODE data at the ENCODE portal. Nucleic Acids Res. 44(D1), D726–D732 26527727 10.1093/nar/gkv1160 PMC4702836

[42] Van Nostrand E. L., Pratt G. A., Shishkin A. A., Gelboin-Burkhart C., Fang M. Y., Sundararaman B., Blue S. M., Nguyen T. B., Surka C., Elkins K., Stanton R., Rigo F., Guttman M., Yeo G. W. (2016) Robust transcriptome-wide discovery of RNA-binding protein binding sites with enhanced CLIP (eCLIP). Nat. Methods 13, 508–514 27018577 10.1038/nmeth.3810 PMC4887338

[43] Aken B. L., Achuthan P., Akanni W., Amode M. R., Bernsdorff F., Bhai J., Billis K., Carvalho-Silva D., Cummins C., Clapham P., Gil L., Girón C. G., Gordon L., Hourlier T., Hunt S. E., Janacek S. H., Juettemann T., Keenan S., Laird M. R., Lavidas I., Maurel T., McLaren W., Moore B., Murphy D. N., Nag R., Newman V., Nuhn M., Ong C. K., Parker A., Patricio M., Riat H. S., Sheppard D., Sparrow H., Taylor K., Thormann A., Vullo A., Walts B., Wilder S. P., Zadissa A., Kostadima M., Martin F. J., Muffato M., Perry E., Ruffier M., Staines D. M., Trevanion S. J., Cunningham F., Yates A., Zerbino D. R., Flicek P. (2017) Ensembl 2017. Nucleic Acids Res. 45(D1), D635–D642 27899575 10.1093/nar/gkw1104 PMC5210575

[44] Smit, A. F. A., Hubley, R., Green, P. (2013-2015) RepeatMasker Open-4.0. Accessed May, 19, 2017, at: http://www.repeatmasker.org

[45] Quinlan A. R., Hall I. M. (2010) BEDTools: a flexible suite of utilities for comparing genomic features. Bioinformatics 26, 841–842 20110278 10.1093/bioinformatics/btq033 PMC2832824

[46] The R Development Core Team (2011) R: A language and environment for statistical computing. The R Foundation for Statistical Computing, Vienna, Austria

[47] Wickham H. (2009) Ggplot2: Elegant Graphics for Data Analysis, Springer, New York

[48] Wilke, C. O. (2016) cowplot - Streamlined plot theme and plot annotations for ggplot2. Accessed on May 19, 2017, at: https://wilkelab.org/cowplot/

[49] Robinson J. T., Thorvaldsdóttir H., Winckler W., Guttman M., Lander E. S., Getz G., Mesirov J. P. (2011) Integrative genomics viewer. Nat. Biotechnol. 29, 24–26 21221095 10.1038/nbt.1754 PMC3346182

[50] Deantonio C., Sedini V., Cesaro P., Quasso F., Cotella D., Persichetti F., Santoro C., Sblattero D. (2014) An Air-Well sparging minifermenter system for high-throughput protein production. Microb. Cell Fact. 13, 132 25218288 10.1186/s12934-014-0132-1 PMC4172861

[51] Schneider C. A., Rasband W. S., Eliceiri K. W. (2012) NIH Image to ImageJ: 25 years of image analysis. Nat. Methods 9, 671–675 22930834 10.1038/nmeth.2089 PMC5554542

[52] Kuwano Y., Pullmann R. Jr., Marasa B. S., Abdelmohsen K., Lee E. K., Yang X., Martindale J. L., Zhan M., Gorospe M. (2010) NF90 selectively represses the translation of target mRNAs bearing an AU-rich signature motif. Nucleic Acids Res. 38, 225–238 19850717 10.1093/nar/gkp861 PMC2800222

[53] Murayama A., Ohmori K., Fujimura A., Minami H., Yasuzawa-Tanaka K., Kuroda T., Oie S., Daitoku H., Okuwaki M., Nagata K., Fukamizu A., Kimura K., Shimizu T., Yanagisawa J. (2008) Epigenetic control of rDNA loci in response to intracellular energy status. Cell 133, 627–639 18485871 10.1016/j.cell.2008.03.030

[54] Schmittgen T. D., Livak K. J. (2008) Analyzing real-time PCR data by the comparative C(T) method. Nat. Protoc. 3, 1101–1108 18546601 10.1038/nprot.2008.73

[55] Wang Y., Zhu W., Levy D. E. (2006) Nuclear and cytoplasmic mRNA quantification by SYBR green based real-time RT-PCR. Methods 39, 356–362 16893657 10.1016/j.ymeth.2006.06.010

[56] Foti R., Zucchelli S., Biagioli M., Roncaglia P., Vilotti S., Calligaris R., Krmac H., Girardini J. E., Del Sal G., Gustincich S. (2010) Parkinson disease-associated DJ-1 is required for the expression of the glial cell line-derived neurotrophic factor receptor RET in human neuroblastoma cells. J. Biol. Chem. 285, 18565–18574 20395301 10.1074/jbc.M109.088294 PMC2881782

[57] Matochko W. L., Cory Li S., Tang S. K., Derda R. (2014) Prospective identification of parasitic sequences in phage display screens. Nucleic Acids Res. 42, 1784–1798 24217917 10.1093/nar/gkt1104 PMC3919620

[58] Castella S., Bernard R., Corno M., Fradin A., Larcher J. C. (2015) Ilf3 and NF90 functions in RNA biology. Wiley Interdiscip. Rev. RNA 6, 243–256 25327818 10.1002/wrna.1270

[59] Lubelsky Y., Ulitsky I. (2018) Sequences enriched in Alu repeats drive nuclear localization of long RNAs in human cells. Nature 555, 107–111 29466324 10.1038/nature25757 PMC6047738

[60] Corthésy B., Kao P. N. (1994) Purification by DNA affinity chromatography of two polypeptides that contact the NF-AT DNA binding site in the interleukin 2 promoter. J. Biol. Chem. 269, 20682–20690 8051169

[61] Kao P. N., Chen L., Brock G., Ng J., Kenny J., Smith A. J., Corthésy B. (1994) Cloning and expression of cyclosporin A- and FK506-sensitive nuclear factor of activated T-cells: NF45 and NF90. J. Biol. Chem. 269, 20691–20699 7519613

[62] Parrott A. M., Mathews M. B. (2007) Novel rapidly evolving hominid RNAs bind nuclear factor 90 and display tissue-restricted distribution. Nucleic Acids Res. 35, 6249–6258 17855395 10.1093/nar/gkm668 PMC2094060

[63] Schmidt T., Knick P., Lilie H., Friedrich S., Golbik R. P., Behrens S. E. (2016) Coordinated action of two double-stranded RNA binding motifs and an RGG motif enables nuclear factor 90 to flexibly target different RNA substrates. Biochemistry 55, 948–959 26795062 10.1021/acs.biochem.5b01072

[64] Jayachandran U., Grey H., Cook A. G. (2016) Nuclear factor 90 uses an ADAR2-like binding mode to recognize specific bases in dsRNA. Nucleic Acids Res. 44, 1924–1936 26712564 10.1093/nar/gkv1508 PMC4770229

[65] Schmidt T., Knick P., Lilie H., Friedrich S., Golbik R. P., Behrens S. E. (2017) The properties of the RNA-binding protein NF90 are considerably modulated by complex formation with NF45. Biochem. J. 474, 259–280 28062840 10.1042/BCJ20160790

[66] Chillón I., Pyle A. M. (2016) Inverted repeat Alu elements in the human lincRNA-p21 adopt a conserved secondary structure that regulates RNA function. Nucleic Acids Res. 44, 9462–9471 27378782 10.1093/nar/gkw599 PMC5100600

[67] Zhang Z., Carmichael G. G. (2001) The fate of dsRNA in the nucleus: a p54(nrb)-containing complex mediates the nuclear retention of promiscuously A-to-I edited RNAs. Cell 106, 465–475 11525732 10.1016/s0092-8674(01)00466-4

[68] Elbarbary R. A., Li W., Tian B., Maquat L. E. (2013) STAU1 binding 3′ UTR IRAlus complements nuclear retention to protect cells from PKR-mediated translational shutdown. Genes Dev. 27, 1495–1510 23824540 10.1101/gad.220962.113 PMC3713430

[69] Kramerov D. A., Vassetzky N. S. (2011) SINEs. Wiley Interdiscip. Rev. RNA 2, 772–786 21976282 10.1002/wrna.91

[70] Kuwano Y., Kim H. H., Abdelmohsen K., Pullmann R. Jr., Martindale J. L., Yang X., Gorospe M. (2008) MKP-1 mRNA stabilization and translational control by RNA-binding proteins HuR and NF90. Mol. Cell. Biol. 28, 4562–4575 18490444 10.1128/MCB.00165-08 PMC2447119

[71] Matsumoto-Taniura N., Pirollet F., Monroe R., Gerace L., Westendorf J. M. (1996) Identification of novel M phase phosphoproteins by expression cloning. Mol. Biol. Cell 7, 1455–1469 8885239 10.1091/mbc.7.9.1455 PMC275994

[72] Parrott A. M., Walsh M. R., Reichman T. W., Mathews M. B. (2005) RNA binding and phosphorylation determine the intracellular distribution of nuclear factors 90 and 110. J. Mol. Biol. 348, 281–293 15811368 10.1016/j.jmb.2005.02.047

[73] Dominissini D., Rechavi G. (2017) 5-methylcytosine mediates nuclear export of mRNA. Cell Res. 27, 717–719 28534483 10.1038/cr.2017.73 PMC5518879

