# Supplementary material for: The RNA-binding protein ILF3 binds to transposable element sequences in SINEUP lncRNAs
Source: FASEB J. 2019 Oct 25;33(12):13572–89. doi: 10.1096/fj.201901618RR (PMC6894054; doi:10.1096/fj.201901618RR)

Supplementary Figure 1. Overview of the RIDome technology platform.

A) The RIDome workflow consists of three key steps: (1) *Selection of interacting ORFs on a target RNA*. Displaying phages are challenged with a biotinylated RNA bait through two cycles of selection and amplification. (2) *Sequencing of the selected inserts and ranking of reads*. ORF inserts are recovered from the selected libraries, sequenced by NGS, ranked and scored according to their frequency. (3) *Recovery and validation of the top ranking ORFs.* High-scoring ORFs are re-cloned from the library by inverse PCR, and their interactions are validated by ELISA or other assays.

Supplementary Figure 2. Single-cycle kinetics analysis of SINEUP RNA – ILF3 interaction. The resulting sensorgram (red line) after double-referencing (subtraction of signal from the reference surface and "zero sample concentration" control cycle), fitted according a 1:1 binding model. Black line represents the theoretical fitted curve.

Supplementary Figure 3. ILF3 binding analysis from ENCODE eCLIP data in human K562 cells. (**A**) SINEs class is the most frequent and enriched class of repeats in overlap with ILF3 peaks. (**B**) The same analysis is carried out on SINEs families (pink) and subfamilies (cyan). Enrichments are measured with respect to the genomic average resulting from randomizations and are appreciable on the Y axes. Larger class/families/subfamilies are on the right. Enrichments are shown as values above zero while depletions are below. (**C**) The numbers of peaks in SINE-containing exons are displayed according to different classes of coding and non-coding exons. (**D**) Organization of the S/AS pair associated to the coding gene UGDH whose antisense contains an embedded inverted AluJb element bound by ILF3 from the eCLIP data. **(E)** Genomic organization of the UGDH locus (Chr4:39,497,200-39,595,745) from the Ensembl genome browser. The track showing mapping of SINEs is in grey whereas ILF3 peaks, loaded as custom track, are in black.

Supplementary Figure 4. ILF3 expression and knocked-down in HEK 293T/17. (**A**) Endogenous ILF3 nuclear localization was assessed by immunofluorescence with anti-DRBP76 (ILF3) antibody in HEK 293T/17 while DJ-1 immunostaining was carried out as control for a non-nuclear enriched protein. (**B**) Endogenous ILF3 was silenced with target specific siRNA and control siRNA in HEK 293T/17.

Supplementary Figure 1

A

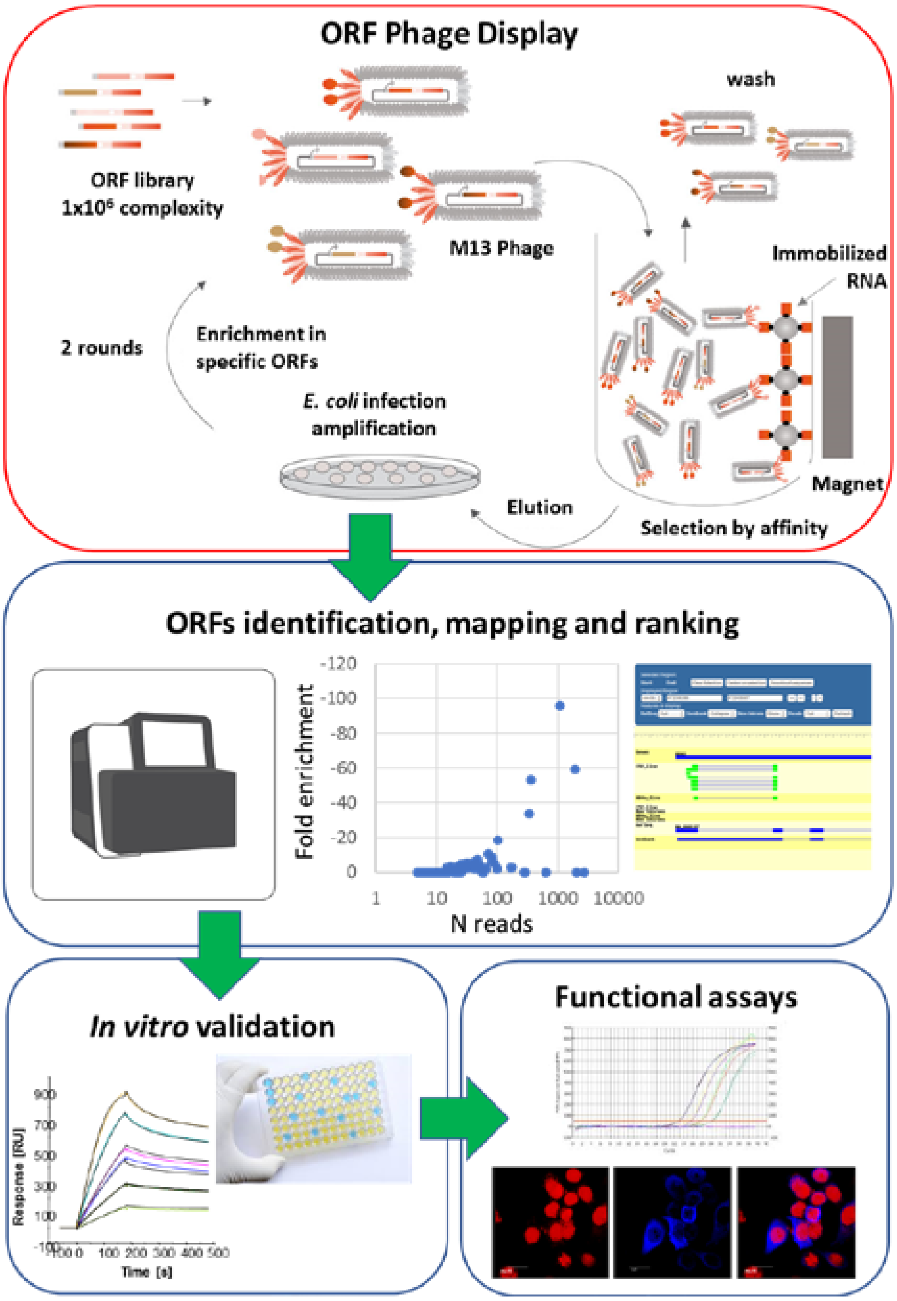

Supplementary Figure 2

A

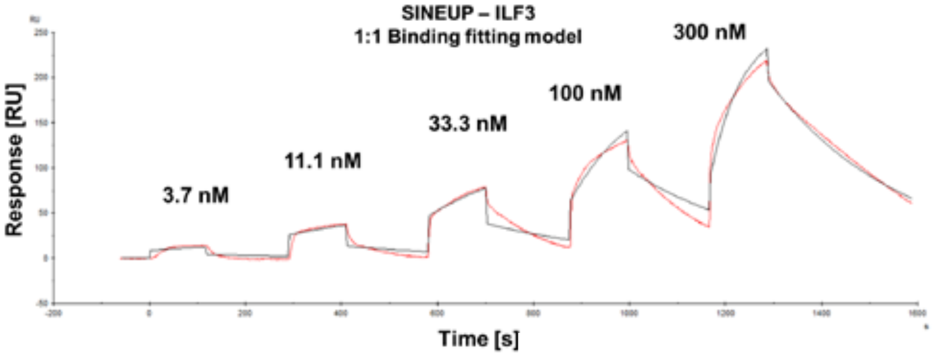

Supplementary Figure 3

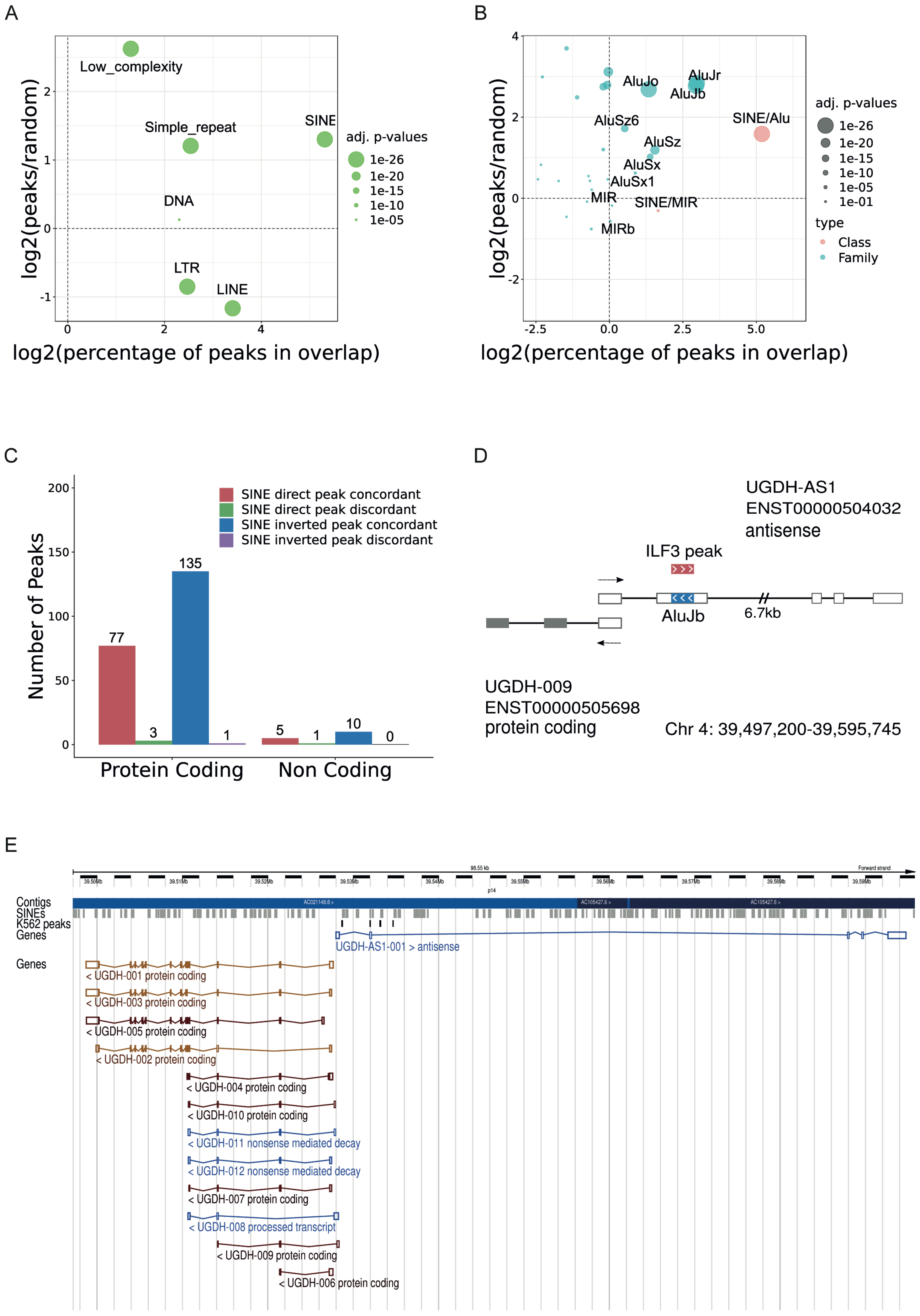

Supplementary Figure 4

A

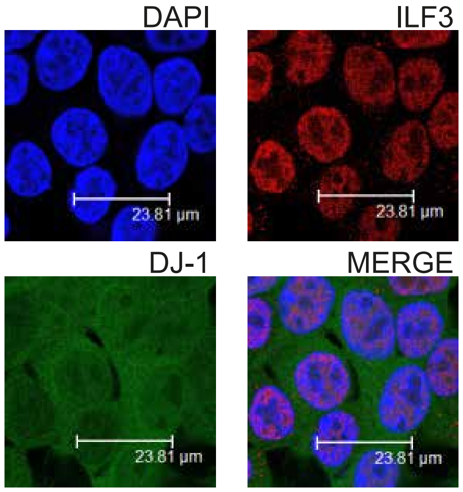

B

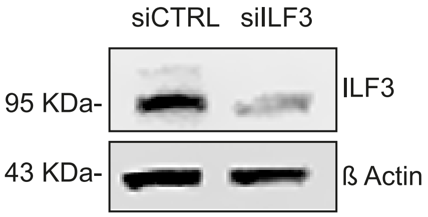

Supplement: Supplementary file 1 [file fj.201901618RR.sd1.pdf]
